# Evaluation of mass spectrometry MS/MS spectra for the presence of isopeptide crosslinked peptides

**DOI:** 10.1371/journal.pone.0254450

**Published:** 2021-07-09

**Authors:** Lawrence M. Schopfer, Seda Onder, Oksana Lockridge

**Affiliations:** 1 Eppley Institute, University of Nebraska Medical Center, Omaha, NE, United States of America; 2 Department of Biochemistry, School of Pharmacy, Hacettepe University, Ankara, Turkey; Aarhus University, DENMARK

## Abstract

Isopeptide crosslinked proteins can be the product of transglutaminase or of exposure to organophosphorus toxicants (OP). Transglutaminase links glutamine to lysine with loss of ammonia. OP toxicants induce a link between glutamic acid and lysine with loss of water. Our goal was to establish criteria to distinguish real from false isopeptide crosslinks reported by software searches of mass spectrometry data. We used fragmentation spectra of tryptic peptides from MAP-rich tubulin *Sus scrofa* as a test system for detection of naturally-occurring isopeptide crosslinks. Data were analyzed with Protein Prospector. Criteria for the assignments included the presence of at least 1 crosslink specific product ion, fragment ions from both peptides, Protein Prospector scores ≥20, and best fit of the MS/MS data to the crosslinked peptide as opposed to a linear peptide. Out of 301,364 spectra, 15 potential transglutaminase-type crosslinked peptide candidates were identified. Manual evaluation of these MS/MS spectra reduced the number to 1 valid crosslink between Q112 of NFH and K368 of Tau. Immunopurification with anti-isopeptide 81D1C2 confirmed that MAP-rich tubulin contained only one isopeptide. Support for this isopeptide bond was obtained by showing that transglutaminase was capable of incorporating dansyl-aminohexyl -QQIV into K368. A model of the KIETHK-QLEAHNR isopeptide was synthesized with the aid of transglutaminase. MS/MS spectra of the model validated our interpretation of the native isopeptide. An OP-induced isopeptide bond between K163 of tubulin alpha-1A and E158 of tubulin beta-4B was induced by treating MAP-rich tubulin with 100 μM chlorpyrifos oxon. This crosslink was supported by the criteria described above and by the presence of diethoxyphospho-lysine 163 in the tubulin alpha-1A peptide. The information obtained in this work is valuable for future studies that aim to understand why exposure to OP is associated with increased risk of neurodegenerative disease.

## Introduction

**Fig 1:**
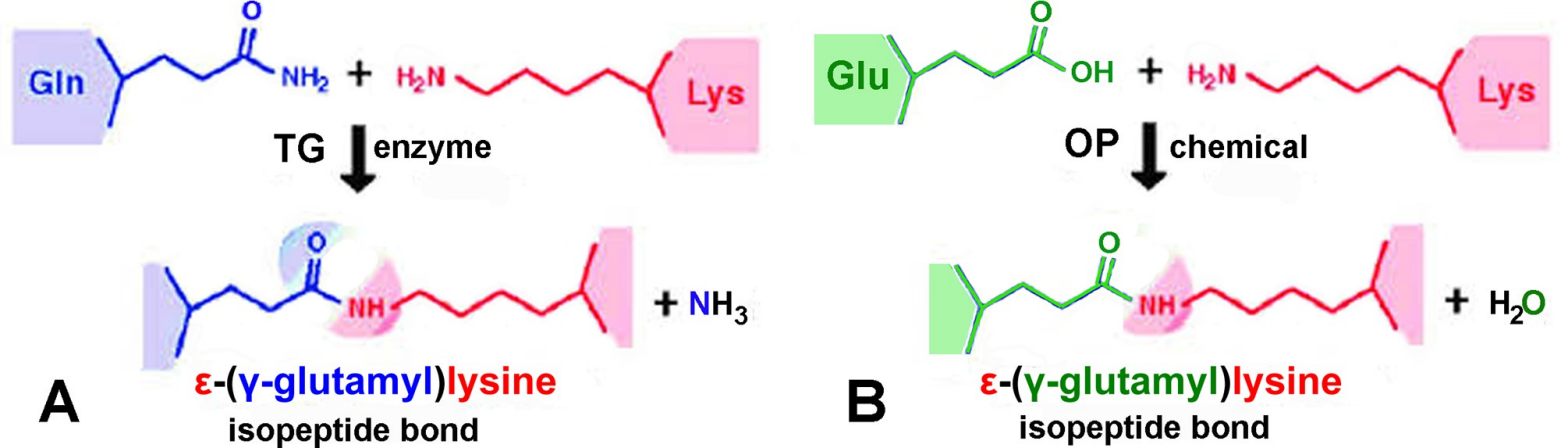
A) Isopeptide bond between glutamine and lysine formed by the action of the transglutaminase (TG) enzyme with release of ammonia. B) Isopeptide bond between glutamic acid (or aspartic acid) and lysine formed by exposure to organophosphorus chemicals (OP) with release of water. Figure adapted from [18].

Transglutaminase (TG) makes a covalent bond between the side chains of glutamine and lysine to yield the protease resistant isopeptide bond and ammonia [1, 2]. See Fig 1A. Organophosphorus pesticides and the nerve agent VX induce a covalent bond between the side chains of glutamic acid (or aspartic acid) and lysine to yield the isopeptide bond and water [3–5]. See Fig 1B. Transglutaminase generates isopeptide crosslinks between proteins that stabilize structures important for brain function [6, 7], neuron differentiation [8], blood coagulation [9], and skin strength [10]. Detrimental effects are also attributed to transglutaminase activity. In neurodegenerative diseases protein aggregates produced by the crosslinking activity of transglutaminase are found in senile plaques, neurofibrillary tangles [11], and Lewy bodies [12]. Transglutaminase-created isopeptide covalent linkages are present in cataracts [13] and celiac disease [14]. Glutamine and lysine residues susceptible to transglutaminase-catalyzed crosslinking are commonly identified by incorporating fluorescent transglutaminase substrates such as dansyl cadaverine and dansyl-aminohexyl QQIV [15] into target proteins. In addition, immunohistochemistry is used for detection of isopeptide crosslinks in tissue sections [16]. In our experience the commercially available anti-isopeptide monoclonal 81D1C2 has poor sensitivity in Western blots as it detects isopeptide bonds only when the number of isopeptide bonds per mg protein is very high. Proteins crosslinked by transglutaminase can be undetectable on a Western blot [17], presumably because the relative number of isopeptide bonds is low.

Use of mass spectrometry for identifying isopeptide crosslinked peptides has been described by several groups [19–22]. The consensus of opinion is that mass spectral identification of crosslinked peptides is fraught with difficulties. A number of strategies have been reported to mitigate these difficulties [22–26]. We adopted elements from these strategies to develop a set of criteria for identifying convincing isopeptide crosslinked peptides. In the present report we use those criteria to identify naturally-occurring KQ isopeptide crosslinked peptides in MAP-rich tubulin protein from *Sus scrofa*. Proteins from MAP-rich tubulin were separated into groups by SDS polyacrylamide gel electrophoresis, extracted from the gel slices, digested with trypsin, and subjected to mass spectrometry. MS/MS spectra were searched for naturally occurring KQ isopeptide crosslinks using Protein Prospector/Batch-Tag software. This was followed by screening of the crosslink candidates using our criteria, and then by manual evaluation of each promising MS/MS spectrum. One crosslinked pair was identified; between Q112 in peptide QLEAHNR from neurofilament heavy polypeptide and K368 in peptide KIETHK from Tau. We generated support for the crosslink by demonstrating that the lysine residue involved in the crosslink was a substrate for reaction with dansyl-aminohexyl QQIV in the presence of transglutaminase.

In a second experiment, we confirmed the presence of this isopeptide crosslinked peptide pair in MAP-rich tubulin by immunopurifying isopeptides on Dynabeads conjugated to anti-isopeptide 81D1C2 antibody. Mass spectrometry data searched with Protein Prospector software identified the isopeptide bond between lysine 368 of Tau and glutamine 112 of neurofilament heavy polypeptide. No additional isopeptides were present in the MAP-rich tubulin preparation. In a third experiment, we crosslinked chemically synthesized peptides with the aid of bacterial transglutaminase and showed that MS/MS spectra of the model isopeptide supported our interpretation of the native isopeptide.

Finally, we applied the process for isopeptide identification to MS/MS spectra of isopeptides created by the crosslinking action of chlorpyrifos oxon. This work will be helpful to future studies aimed at understanding the association between OP pesticide exposure and neurodegenerative disease [27].

## Methods and materials

### Materials

MAP-rich tubulin *Sus scrofa* from porcine brain, Cytoskeleton Inc. ML116, stored at -80˚C.

Trypsin, Promega, Sequencing grade, cat# V5113, stored at -80˚C.

Dansyl cadaverine 20 mM in dimethylsulfoxide stored at -20˚C, Fluka 30432.

Dansyl-aminohexyl QQIV 22 mM in dimethylsulfoxide stored at -20˚C, Zedira GmbH D001.

Recombinant human transglutaminase TG2 produced in E. coli, Zedira GmbH T002. Aliquots of 1 mg/mL (3 U/μL) were stored at -20˚C.

Recombinant microbial (bacterial) transglutaminase, Zedira GmbH T001, UniProt P81453. The lyophilized powder was reconstituted with water to 4.5 mg/mL, 32 U/mg.

Chlorpyrifos oxon CAS:5598-15-2 Chem Service Inc. MET-11459B stock solution in acetonitrile stored at -80˚C.

Dynabeads-Protein G, Novex by Life Technologies AS, Oslo, 30 mg/ml

Mouse anti-isopeptide monoclonal 81D1C2, IgG1 kappa, LSBio LS-C153331

Dimethyl pimelimidate dihydrochloride, Sigma D8388.

Synthetic peptides 95% pure, Genscript Inc.

### SDS gel electrophoresis

Samples of MAP-rich tubulin were reduced and denatured with dithiothreitol/SDS in a boiling water bath for 3 min. Gradient 4–22% polyacrylamide gels were prepared in a Hoefer Scientific vertical slab gel SE600 system. Electrophoresis was at 200 volts constant voltage for 7 hours at room temperature. Gels were stained with Coomassie blue.

### Gel slices for analysis of KQ crosslinked peptides in MAP-rich tubulin

Peptides from an abundant protein can suppress mass spectrometry detection of peptides from less abundant proteins. In the case of MAP-rich tubulin, the abundant tubulin peptides can suppress detection of microtubule associated peptides. We overcame this problem by separating proteins on an SDS polyacrylamide gel, staining with Coomassie blue, cutting the visible bands from the gel, and analyzing tryptic peptides extracted from gel slices. Gel slices were taken from 5 SDS gels, ranging from 15 kDa to the top of the separating gel. Three separate samples were prepared. A total of 29 gel slices were analyzed. Proteins in each of the 29 gel slices were reduced with dithiothreitol, alkylated with iodoacetamide, and digested with trypsin. Peptides were extracted in preparation for LC-MS/MS using the method described by Peeples et al. [28].

### Conjugation of anti-isopeptide monoclonal to Dynabeads-Protein G

Mouse anti-isopeptide monoclonal 81D1C2 was conjugated to Dynabeads-Protein G using the Sporty et al. [29] protocol. A 100 μL suspension of Dynabeads-Protein G was transferred to a microfuge tube, where the magnetic beads were washed 3 times with phosphate buffered saline (PBS). A 20 μg aliquot (20 μL) of antibody and 380 μL of PBS were added to the washed 3 mg beads to make a 0.05 mg/mL antibody solution. The sample was rotated overnight at room temperature to allow the antibody to bind to Protein G. The supernatant was discarded and the beads were washed 2 times with 0.2 M triethanolamine hydrochloride pH 7.8, 0.02% (w/v) sodium azide. A freshly prepared 200 μL solution of 0.010 g dimethyl pimelimidate dihydrochloride in 1.8 mL of 0.2 M triethanolamine pH 7.8, 0.02% azide was added to the beads. The tube was rotated 30 min at room temperature. After 30 min, the liquid was discarded. The conjugation reaction was quenched by incubating the beads with 200 μL of 20 mM TrisCl, 0.15 M NaCl for 15 min. The beads were washed 3 times with 200 μL of 20 mM TrisCl, 0.15 M NaCl containing 0.05% (v/v) of Tween-20. The washed beads were used immediately for immunopurification of isopeptides.

### Immunopurification of isopeptides from a tryptic digest of MAP-rich tubulin

MAP-rich tubulin (100 μg) in 0.2 mL of 20 mM TrisCl pH 8.5, 0.01% azide (w/v) was treated with 10 mM dithiothreitol for 3 min in a boiling water bath, followed by alkylation of sulfhydryl groups with 50 mM iodoacetamide for 30 min in the dark. The sample was diluted with water to 0.5 mL. The 0.5 mL sample was injected into a hydrated Slide-A-Lyzer cassette 7,000 MWCO Pierce 66373. After 48 hours of dialysis against 2 x 4 L of 20 mM ammonium bicarbonate at 4˚C, the desalted MAP-rich tubulin was recovered in 0.45 mL of 20 mM ammonium bicarbonate. The carbamidomethylated MAP-rich tubulin was digested with 2 μg trypsin overnight at 37˚C. Trypsin was heat inactivated in a boiling water bath for 5 min.

The 450 μL of carbamidomethylated, desalted tryptic peptides were added to 3 mg Dynabeads-Protein G-81D1C2 and rotated overnight at room temperature. The beads were washed 3 times with phosphate buffered saline, and 2 times with water. Bound peptides were released with 50 μl of 1% (v/v) formic acid pH 2, followed by a second extraction with 50 μl of 1% formic acid. The extracted peptides were dried in a vacuum centrifuge. The dry peptides were dissolved in 20 μL water. The sample was centrifuged at 15,300xg for 20 min before the top 10 μL were transferred to an autosampler vial for mass spectrometry analysis.

### Transglutaminase-catalyzed incorporation of dansyl cadaverine and dansyl-aminohexyl QQIV into MAP-rich tubulin

Solutions of MAP-rich tubulin (1 mg/mL) in 0.1 mL of 20 mM TrisCl pH 8.5 containing 2.5 mM calcium chloride, 1 mM dithiothreitol and 1 mM dansyl cadaverine or 1 mM dansyl-aminohexyl QQIV were incubated with 3 μL of 3 U/μL human transglutaminase for 16 h at 37˚C. Proteins in the reaction mixture were separated on SDS gels and stained with Coomassie blue. Seven bands were cut from the gel at about 15, 30, 50, 90, and 150 kDa plus the top of the separating gel and the top of the stacking gel. Proteins in gel slices were digested with trypsin and prepared for liquid chromatography tandem mass spectrometry as described [28].

### Synthesis of 1605 Da isopeptide

Our goal was to synthesize a model crosslinked dipeptide composed of KIETHK linked to QLEAHNR through a KQ isopeptide bond. The model dipeptide could be used to validate the MS/MS spectrum of the dipeptide from MAP-rich tubulin. The plan was to link 2 precursor peptides through the crosslinking action of bacterial transglutaminase, followed by digestion with trypsin to yield the desired KQ dipeptide. Genscript Inc. synthesized precursor peptides RFAGYIDKVRQLEAHNR sample name NFH and ITHVPGGGNRKIETHK sample name Tau. The chemically synthesized peptides, 2 mg each in 200 μL of 20 mM imidazole pH 7.5, were combined into one vial. The pH of the 400 μL peptide solution was raised from 6 to 7 by addition of 4 μL of 1 M sodium hydroxide. The crosslinking reaction was catalyzed by 6 μL of bacterial transglutaminase (27 μg) for 16 h at 37˚C. MALDI-TOF mass spectrometry showed a single peak at the predicted mass of the crosslinked construct, 3800 Da, suggesting that 100% of the peptides had been crosslinked. A 10 μL aliquot containing 50 μg of 3800 Da crosslinked peptide was diluted to 50 μL with water and digested with 2 μg trypsin. The expected peak at 1605 Da, for the KIETHK_QLEAHNR crosslinked product was present at low abundance. The digest was enriched for the 1605 Da peptide by high performance liquid chromatography on a C18 Phenomenex column Prodigy 5 μ ODS(2), 11x 4.60 mm, part no.00D-3300-EO where the 1605 Da peptide eluted in 16% acetonitrile, 0.1% trifluoroacetic acid. The fraction containing the 1605 Da peptide was subjected to LC-MS/MS on the Orbitrap mass spectrometer.

### Bruker autoflex maX MALDI-TOF/TOF

Fractions eluted from the HPLC separation of crosslinked synthetic peptides were screened for the presence of the 1605 Da parent ion by spotting 2 μL samples on the target plate with α-cyano-4-hydroxycinnamic acid matrix. MS/MS spectra were acquired using the LIFT method.

### Incubation of MAP-rich tubulin with 100 μM chlorpyrifos oxon

A 0.2 mL solution of 0.5 mg/mL MAP-rich tubulin in 20 mM TrisCl pH 8.5, 0.01% azide was incubated with 2 μL of 10 mM chlorpyrifos oxon (dissolved in acetonitrile) to give a final concentration of 100 μM chlorpyrifos oxon. After 48 hours at 37˚C, the sample was diluted to 1 mL with 20 mM ammonium bicarbonate pH 8 and injected into a hydrated Slide-A-Lyzer Dialysis Cassette #66370 Thermo Scientific 7,000 MWCO. The sample was dialyzed against 2 x 450 mL of 20 mM ammonium bicarbonate pH 8 at 4˚C to remove excess chlorpyrifos oxon. The dialyzed sample was concentrated to 50 μL in a vacuum centrifuge before 70 μg were loaded on an SDS gel. Coomassie blue stained bands were cut out of the gel. Proteins extracted from gel slices were reduced with dithiothreitol, alkylated with iodoacetamide, and digested with trypsin as described [28] in preparation for liquid chromatography tandem mass spectrometry.

### In-solution sample preparation for liquid chromatography tandem mass spectrometry (LC-MS/MS)

MAP-rich tubulin solutions (0.2 mL in 20 mM TrisCl pH 8.5, 0.01% azide at 0.5 mg/mL) were diluted to 1 mL before the proteins were reduced with 10 mM dithiothreitol in a boiling water bath for 3 min, alkylated with 50 mM iodoacetamide, and dialyzed against 20 mM ammonium bicarbonate pH 8 to remove salts. The volume of the carbamidomethylated proteins was reduced from 1 mL to 0.05 mL in a vacuum centrifuge. Protein concentration, estimated from absorbance at 280 nm in a NanoDrop spectrophotometer (Thermo Fisher Scientific), indicated a recovery of about 65%. Protein at a concentration of about 1.3 μg/μL in 50 μL of 20 mM ammonium bicarbonate was digested with 1 μg trypsin at 37˚C for 16 h. Trypsin was inactivated by addition of formic acid to 0.1%. Particles that could plug the narrow tubing in the liquid chromatography system were removed by centrifuging the samples for 30 min at 14,000 x g in a microfuge. The top 15 μL were transferred to autosampler vials for liquid chromatography tandem mass spectrometry (LC-MS/MS). The protein concentration in the 15 μL supernatant ranged from 0.5 to 0.9 μg/μL.

### Orbitrap Fusion Lumos Tribrid mass spectrometer

Peptide separation was performed with a Thermo RSLC Ultimate 3000 ultra-high pressure liquid chromatography system (Thermo Scientific) at 36°C. Solvent A was 0.1% formic acid in water, and solvent B was 0.1% formic acid in 80% acetonitrile. Peptides were loaded onto an Acclaim PepMap 100 C18 trap column (75 μm x 2 cm; Thermo Scientific cat# 165535) at a flow rate of 4 μL/min and washed with 100% solvent A for 10 minutes. Then, they were transferred to a Thermo Easy-Spray PepMap RSLC C18 column (75 μm x 50 cm with 2 μm particles, Thermo Scientific cat# ES803) and separated at a flow rate of 300 nL/min using a linear gradient from 5 to 50% solvent B in 20 min, followed by another linear gradient from 50 to 100% solvent B in 40 minutes. The column was washed with 100% solvent B for 30 minutes before being re-equilibrated with 5% solvent B for 25 minutes.

Eluted peptides were sprayed directly into a Thermo Orbitrap Fusion Lumos Tribrid mass spectrometer (Thermo Scientific). Data were collected using data dependent acquisition. A survey full scan MS (from 350–1800 m/z) was acquired in the Orbitrap with a resolution of 120,000. The AGC target (Automatic Gain Control for setting the ion population in the Orbitrap before collecting the MS) was set at 4 x 10^5^ and the ion filling time was set at 100 msec. The 25 most intense ions with charge state of 2–6 were isolated in a 3 sec cycle and fragmented using HCD (high-energy collision induced dissociation) with 35% normalized collision energy. Fragment ions were detected in the Orbitrap with a mass resolution of 30,000 at 200 m/z. The AGC target for MS/MS was set at 5 x 10^4^, ion filling time at 60 ms, and dynamic exclusion at 30 sec with a 10 ppm mass window. Data were reported in *.raw format.

### Proteins identified in MAP-rich tubulin *Sus scrofa*

Protein identification was performed on an in-solution tryptic digest of a MAP-rich tubulin sample with Proteome Discoverer v 2.4 (Thermo Scientific, Waltham MA) using a Sequest HT search engine. MS/MS data were searched against an NCBI, *Sus scrofa* protein database (downloaded June 2020). The search was set-up for full tryptic peptides with a maximum of two missed cleavage sites. Oxidized methionine was included as a variable modification. Carbamidomethylation of cysteine was set as a fixed modification. The precursor mass tolerance threshold was set at 10 ppm with a fragment tolerance of 0.02 Da. The significance threshold of the ion score was calculated based on a false discovery rate of ≤ 1% using Protein FDR Validator with Posterior Error Probabilities provided by the Percolator algorithm associated with the Sequest HT search engine.

### Search for KQ crosslinked peptides with Batch-Tag software in protein prospector version 6.2.1

The *.raw data files from the Orbitrap Fusion Lumos Tribrid were converted to *.mgf files using MSConvert (ProteoWizard Tools from SourceForge). The *.mgf files were analyzed using Batch-Tag Web [24] on the Protein Prospector website https://prospector.ucsf.edu [prospector.ucsf.edu]

We created a specialized user database that included five abundant proteins from those identified in the *Sus scrofa* MAP-rich tubulin sample, plus neurofilament heavy polypeptide (see S1 Table). The six proteins were: tubulin alpha-1A chain (NP_001302639), tubulin beta-4B chain (XP_003122400), microtubule-associated protein 2 isoform X8 (XP_013839898), microtubule-associated protein 1B isoform X1 (XP_003134080), microtubule-associated protein Tau isoform X16 (XP_020922473), and neurofilament heavy polypeptide isoform (XP_005670835). Sequences were pasted into the User Protein Sequence window of Batch-Tag Web in FASTA format. MS/MS data were searched against this 6-protein database with Batch-Tag Web for isopeptide crosslinks between lysine (K) and glutamine (Q).

The search parameters were as follows. 1) Database: User protein. 2) User Protein Sequences: FASTA files from NCBI Protein Database for the user proteins were pasted into this window. 3) Precursor Charge Range: 2 3 4 5. 4) Masses: monoisotopic. 5) Parent Tol: 20 ppm. Frag Tol: 30 ppm. 6) Instrument: ESI-Q-high-res. 7) Digest: Trypsin. 8) Max missed cleavages: 3. 9) Constant Mods: Carbamidomethyl (C). 10) Variable Mods: Oxidation (M). 11) Expectation Calc Method: None. 12) Mass Modifications: range -18 to 3883 Da. (Formation of the isopeptide bond between K and Q is accompanied by loss of 17 Da due to loss of ammonia. The -18 mass modification allows for loss of water (-18) and loss of ammonia (-17). 13) Check mark in boxes K and Q. 14) Check mark in box Uncleaved. Checking the Uncleaved box avoids false candidates in which a C-terminal lysine is reported as the crosslinked lysine. Such reports are false because trypsin does not cleave modified lysines. 15) Crosslinking; Link Search Type: User Defined Link. 16) User Defined Link Parameters; Link AAs: K, Protein N-term>Q. 17) Bridge Elem Comp: N-1 H-3. S1 Fig is a screen shot of the Batch-Tag Web page showing the search parameters for KQ crosslinked peptides.

Protein Prospector identifies peptide crosslinks via a mass modification process wherein the mass of one peptide plus the crosslink mass is considered as a modification of the other peptide. This is an efficient process that minimizes the search space, but it does not recognize masses that contain fragments of both peptides, or fragment masses that involve unexpected amino acid modifications. Identifying these masses requires manual inspection of the fragmentation pattern.

### Protein prospector search for KE and KD crosslinked peptides

Parameters used on the Batch-Tag Web page to search for KE and KD crosslinked peptides were as follows. Parameters 1) to 12), 14) and 15) were identical to those in the search for KQ crosslinked peptides above. 13) Check mark in boxes D, E, and K. 16) User Defined Link Parameters; Link AAs: E, D, Protein C-term>K, Protein N-term. 17) Bridge Elem Comp: H-2 O-1.

### Protein prospector/search compare and Xcalibur/qual browser

Upon completion of a search, a Search Compare page appears. Selecting Report Type: Crosslinked Peptides yields the “Search Compare Search Results” page listing the crosslinked peptide results from the Batch-Tag search. This page contains sequences of candidate crosslinked peptides, identifies the crosslinked residues, shows a score and score difference for each crosslinked pair, and other useful parameters. It also provides a link to an MS/MS spectrum for the crosslinked peptides that includes a list of observed and theoretical fragment masses, and a matched intensity score [24]. Protein Prospector scores and score differences are based on the number and types of fragment ions identified, as well as their sequence and charge [24]. Crosslinked candidates from the Search Compare page were screened using the criteria described in sections “Features of an MS/MS spectrum that support an isopeptide crosslink” and “Features of an MS/MS spectrum that raise doubts about a candidate crosslinked peptide pair” from Results.

We also evaluated the crosslinked peptides reported on the “Search Compare Search Results” page with the aid of MS-TAG, a program in Protein Prospector to test for linear peptides that fit the data better than the crosslinked candidate. For MS-Tag search details refer to the section below entitled “MS-Tag search for alternative interpretations”.

We used Xcalibur/Qual Browser (Thermo Scientific, Waltham MA) to manually evaluate each crosslinked candidate. Manual evaluation included identifying ions consistent with the crosslinked peptide sequence, and identifying ions that could fit other sequences that were not identified by Protein Prospector. The S1 Text file describes details of our manual evaluation protocol.

### Protein prospector search for dansyl cadaverine adducts on glutamine

Parameters used on the Batch-Tag Web page to search for dansyl cadaverine adducts on glutamine were as follows. 1) to 11) and 13) were identical to those in the search for KQ crosslinked peptides above. 12) Mass Modifications: range 318 to 320 Da. Check mark in box Q. 14) Crosslinking; Link Search Type: No Link. 15) User Defined Variable Modifications: Mod 1 Label: dansyl cadaverine; Specificity: Q; Mod 1 Elem Comp: C17H22N2O2S.

### Protein prospector search for dansyl-aminohexyl QQIV adducts on lysine

Parameters used on the Batch-Tag Web page to search for dansyl-aminohexyl QQIV adducts on lysine were as follows. Parameters 1) to 11), 13) and 14) were identical to parameters in the search for dansyl cadaverine adducts above. 12) Mass Modifications: range 815 to 816 Da. Check mark in box K. 15) User Defined Variable Modifications; Mod 1 Label: dansylQQIV; Specificity: K; Mod 1 Elem Comp: C39H57N7O10S.

### MS-tag search for alternative interpretations

Potential crosslinked peptides were checked for the possibility that the data fit better to a linear peptide than to a crosslinked peptide. One indication that a linear peptide is a better fit is seeing that one peptide is matched with all b-ions and the other with all y-ions. A second indication is the absence of fragments specific for the shorter peptide. Searches for linear peptides were made with the MS-Tag feature of Protein Prospector. Access to MS-Tag is made via the Search Compare Search Results page in Protein Prospector. This page lists the retention time for each potential crosslink product. A click on the retention time (RT) brings up the MS-Tag page. MS-Tag provides an efficient means for searching a selected MS/MS data set against a large database. We searched each spectrum 3 times. The variables were 1) database NCBInr 2013.6.17 and Taxonomy All species, 2) database NCBInr 2013.6.17 and Taxonomy *Sus scrofa*, and 3) 6-user protein database and Taxonomy *Sus Scrofa*. A constant parameter in each search was Crosslinking> User Defined Link: No Link. The No Link setting instructed the software to search for linear peptides that fit the MS/MS data. Searching All species provided a means of finding unexpected linear fits such as human keratin.

## Results

### Proteins in MAP-rich tubulin *Sus Scrofa*

Mass spectrometry data for in-solution, trypsin-digested MAP-rich tubulin from *Sus scrofa* were searched against an NCBInr 2020.6 *Sus scrofa* database for proteins. Searching against the UniProt database was not an option because the *Sus scrofa* entries are very limited in the UniProt database. At least 2 peptide spectral matches (PSM) were found for 111 proteins, see S1 Table. The most abundant protein, tubulin alpha-1A, was identified by 1115 PSM. The most abundant microtubule-associated protein, MAP2 X8, was identified by 315 PSM. Tubulin peptides accounted for 85% of the total peptides, while microtubule-associated proteins accounted for 11%. Other proteins accounted for 4%. This estimate is similar to the ratio reported by Cytoskeleton Inc. for MAP-rich tubulin *Sus scrofa*. Neurofibrillary heavy polypeptide (NFH) was represented by 2 PSM. Additional NFH peptides were found in digests of gel slices.

### SDS gel electrophoresis

**Fig 2:**
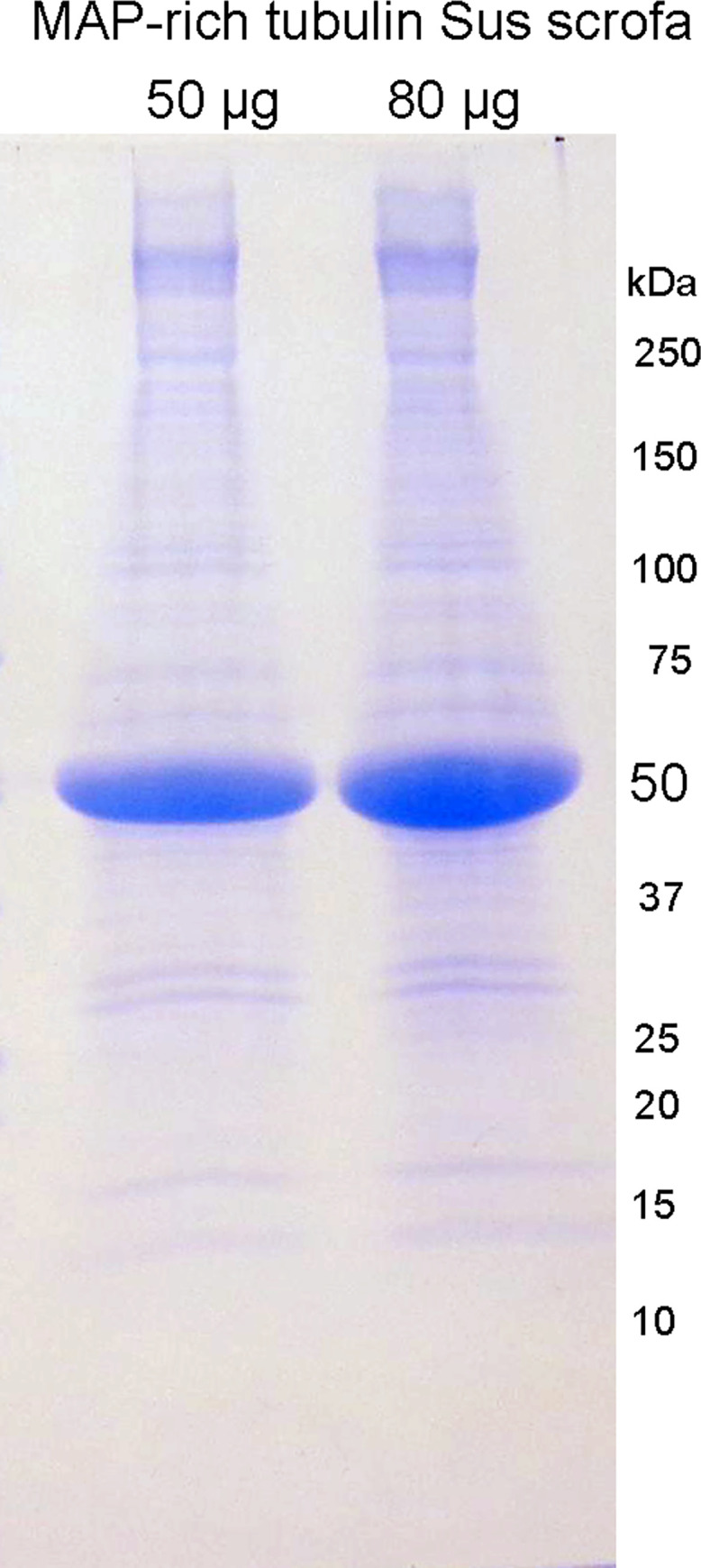
SDS gel stained with Coomassie blue. The intense blue band at 50 kDa is tubulin. Tubulin accounts for about 85% of the protein in MAP-rich tubulin *Sus scrofa*. Bands near the top of the gel include microtubule-associated proteins.

The abundance of tubulin relative to other proteins in the MAP-rich tubulin preparation is visualized in the Coomassie stained SDS gel in Fig 2. The tubulin proteins at 50 kDa have the most intense band. The gel confirms that tubulin accounts for about 85% of the protein in the MAP-rich tubulin preparation.

### Features of an MS/MS spectrum that support an isopeptide crosslink

We used the following set of features to identify isopeptide crosslinked peptide candidates. These features could be applied to any type of crosslinked peptide. An important initial requirement is to collect the data at high mass accuracy and at good resolution, e.g. the Orbitrap Fusion Lumos Tribrid has a mass accuracy of <3 ppm, a parent ion resolution of 120,000, and a fragment ion resolution of 30,000.

Each candidate KQ isopeptide-crosslinked peptide pair was checked for the following features: a) Each peptide in the crosslink contains at least 4 amino acids. b) At least 1 fragment ion is a crosslink specific product ion, that is, it includes the isopeptide link between K and Q. c) The crosslink specific product ions have an intensity greater than 2-fold above background. d) Though one peptide in the fragmentation spectrum generally exhibits most of the product ions, the second peptide must also contribute product ions. This is indicated by a score difference value greater than zero. e) The total Batch Tag/Search Compare score (i.e. the score plus the score difference) of the crosslinked product is greater than 20 [24]. f) The crosslink is on an internal lysine corresponding to a missed tryptic cleavage site. Labeled lysines at the C-terminus of the peptide normally are not accepted because lysine modification blocks tryptic digestion. g) The error for assigned fragment ions is less than 30 ppm when the data are taken with an ESI-Q-high resolution mass spectrometer, e.g. an Orbitrap Fusion Lumos Tribrid. h) The data fit the crosslink product better than a linear peptide. i) Peaks representing greater than 40% of the intensity in the spectrum should be assigned to the crosslinked peptides. This is referred to as % matched intensity. j) For KQ crosslinks, between peptides from the same protein, it is necessary to insure that the peptides are not contiguous. If they are, and if there is a deamidation modification, a net loss of 17 Da would ensue, invalidating a crosslink assignment. However, other interpretations are possible: a linear peptide could be internally crosslinked as illustrated in Fig 9 of the section “Isopeptide crosslink in a linear peptide sequence” in the Discussion.

Of these features, score difference is the best indicator for the presence of a crosslink. Combining score difference with a high % matched intensity provides an even better indication of a crosslink [24].

For KE or KD crosslinked peptide candidates there is a consideration similar to that in item j) above. Formation of a KE or KD isopeptide bonds involves loss of water (18 Da). Loss of water also occurs when a normal peptide bond is formed. Therefore, when both peptides in a KE or KD crosslinked candidate are from the same protein, it is necessary to insure that the peptides are not contiguous in the linear protein sequence.

### Features of an MS/MS spectrum that raise doubts about a candidate isopeptide crosslinked peptide pair

Certain features of an MS/MS spectrum raise doubts about a candidate crosslinked-peptide pair. A fragmentation spectrum is not acceptable as a crosslinked product if: a) All the critically important crosslink specific product ions have an intensity less than 2-fold above background. b) The charge state of the crosslinked peptide pair is +6 or greater. c) One of the peptides includes the N-terminal leader sequence; leader sequences are usually not found in secreted proteins. d) Fragment ions from only one peptide are present. e) Most crosslink specific product ions fit a peptide sequence that is not part of the crosslinked pair. f) The total score of the crosslinked product is less than 20 or the score difference is zero. g) The matched intensity is less than 40%. h) The crosslink involves the C-terminal lysine. Lysines that appear to be labeled at the C-terminus of the peptide are normally not accepted because it is generally found that lysine modification blocks tryptic fragmentation at the C-terminus. However, this does not seem to be true always. There are reports of tryptic cleavage at the C-terminal lysine for ubiquitinated proteins that are modified by an isopeptide linkage to GlyGly [30] and of tryptic cleavage at C-terminal dimethylated lysine [31].

### Naturally-occurring crosslinked proteins in MAP-rich tubulin *Sus scrofa*

The 29 gel slices plus an in-solution digest of MAP-rich tubulin, yielded a total of 301,364 fragmentation spectra. The Protein Prospector search of these 301,364 spectra for crosslinked peptides resulted in 802 potential KQ crosslinked peptides when the search tolerances were set to 20 ppm for the parent and 30 ppm for fragment ions. Application of the features for identifying crosslinked peptides reduced the list to 15 possible crosslinked peptides. Manual evaluation of these 15 MS/MS spectra resulted in 1 convincing crosslinked spectrum. The naturally-occurring crosslink was between Q112 in peptide QLEAHNR of NFH and K368 in peptide KIETHK of Tau. This crosslinked peptide was identified in 5 separate gel slices of MAP-rich tubulin *Sus scrofa*.

Reviewers suggested searching the files with more stringent parameters to reduce the number of crosslinked candidates. In Table 1 we searched the files of 5 separate gel slices that yielded the QLEAHNR/KIETHK crosslinked peptide. The files were searched with parent and fragment ion tolerances ranging from 20/30 ppm to 5/5 ppm.

**Table 1:** Relationship between search tolerance and number of xlink hits.

Parent/frag tolerance	# gel slices with real xlink	# high score >20	# correct xlinks	Scores for correct xlink
20/30 ppm	5	92	7	16.0, 16.3, 20.1, 21.8, 22.3, 27.0 30.1
10/10 ppm	4	42	5	16.6, 17.2, 20.5, 20.7, 27.2
5/10	0	26	0	-
5/5	0	16	0	-

The QLEAHNR/KIETHK crosslink was found in 5 gel slices searched with tolerances 20 and 30 ppm, in 4 gel slices searched with tolerances 10 and 10 ppm, and in zero gel slices searched with tolerances 5 and 10 ppm or 5 and 5 ppm. The scores for the correct crosslinked peptide pair ranged from 16.0 to 30.1. Crosslinked peptides were found in two charge states, thus explaining why 5 gel slices yielded 7 crosslinked peptides, and why 4 gel slices yielded 5 crosslinked peptides. False positives are defined as the difference between #high score and #correct xlinks. Searches conducted with parent and fragment tolerances of 10/10 ppm reduced the number of false positives without losing the correct crosslinked peptide pair.

**Fig 3:**
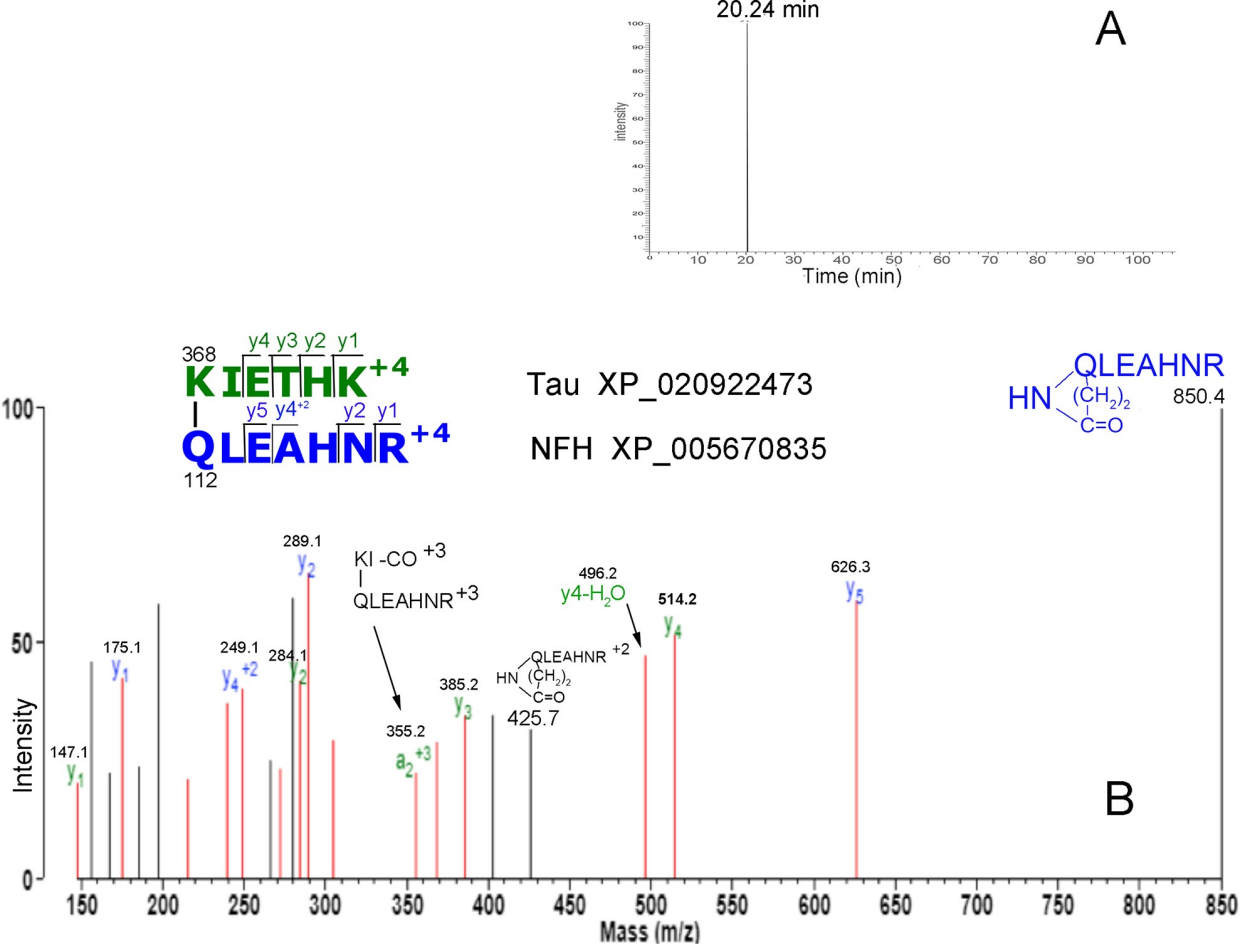
Immunopurified isopeptide from MAP-rich tubulin. Panel A shows that immunopurification on Dynabeads-Protein G-anti-isopeptide 81D1C2 yielded a single peptide eluting at 20.24 min. Panel B is the MS/MS spectrum of the immunopurified, naturally-occurring isopeptide in MAP-rich tubulin. The crosslink is between K368 of Tau and Q112 of NFH. The quadruply-charged parent ion is at 401.96 m/z.

A second method was used to find crosslinked peptides in MAP-rich tubulin. Peptides from an in-solution tryptic digest of MAP-rich tubulin were immunopurified by binding to immobilized anti-isopeptide monoclonal 81D1C2. Only one crosslinked peptide was found, QLEAHNR_KIETHK. Fig 3A shows a single peak, eluting at 20.24 min from the C18 column in the LC-MS/MS system. The MS/MS spectrum in Fig 3B identifies this peak as the dipeptide containing an isopeptide bond between K368 of Tau and Q112 of NFH. The MS/MS spectrum for the crosslinked peptide identified in a gel slice is S4 Fig.

### Manual evaluation

Manual evaluation is a critical step in the process of identifying crosslinked peptides. The S1 Text provides guidance on the procedures for manual evaluation. S2 Table lists the mass differences for dehydro-amino acid residues in charge states +1, +2, and +3. S2 and S3 Figs give an example of a candidate crosslinked peptide that on first inspection looked real, but was rejected as a false positive following manual evaluation.

### Methods to confirm the assignment of isopeptide crosslinked peptides

**Fig 4:**
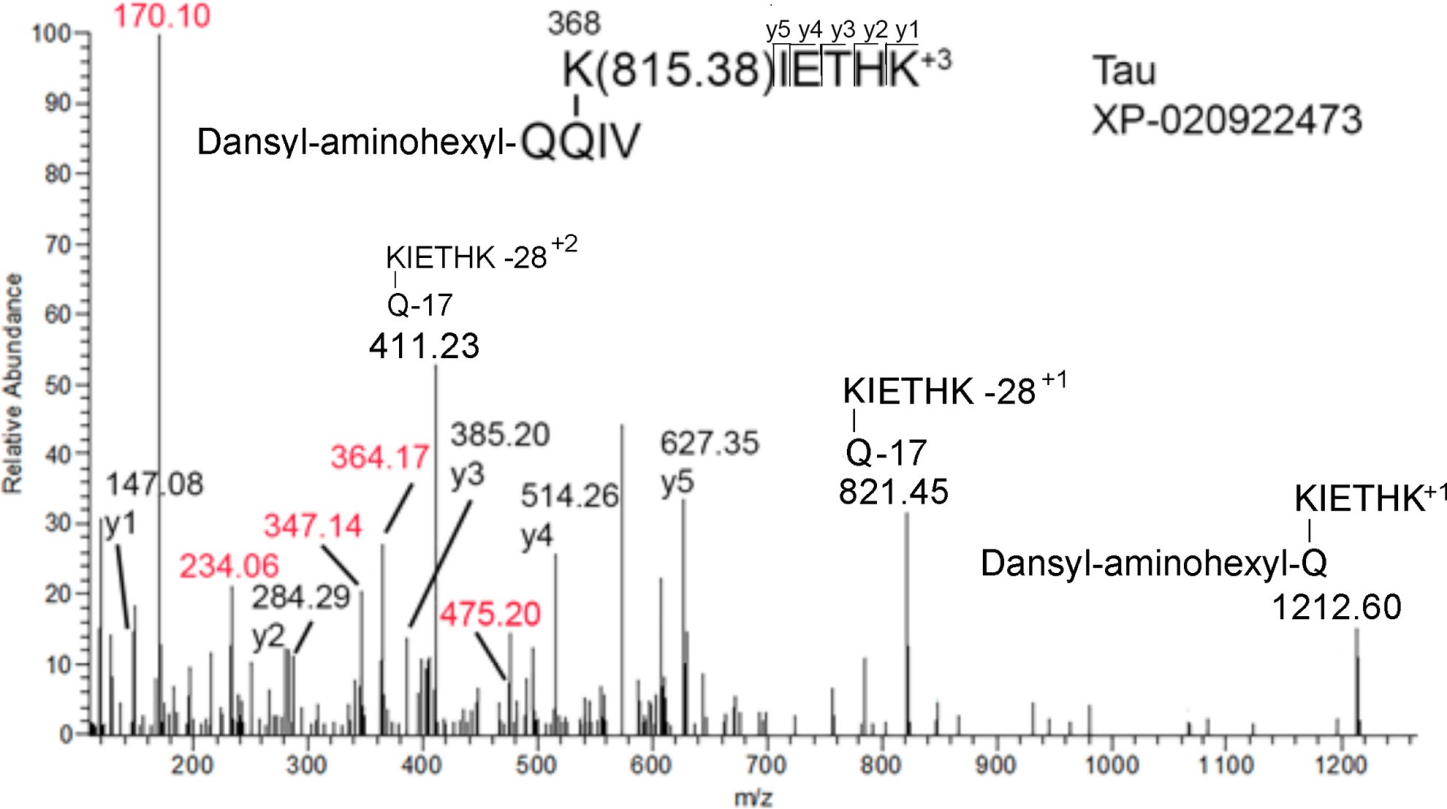
MS/MS spectrum of the dansyl-aminohexyl-QQIV adduct at K368 of Tau. This peptide was found in a gel slice from MAP-rich tubulin *Sus scrofa* that had been treated with dansyl-aminohexyl-QQIV and human transglutaminase. A complete y-ion sequence was obtained from the triply-charged parent ion, at 524.28 m/z. Characteristic ions from the fragmentation of dansyl-aminohexyl QQIV were observed at 170.10, 234.06, 347.14, 364.17, and 475.20 (shown in red) [35]. Linkage between the peptide lysine and the adduct glutamine occurs at both glutamines in Q_1_Q_2_IV. The ion at 475.20 is evidence for linkage to Q_2_. The ion at 1212.60 is evidence for linkage to Q_1_.

To support the identification of crosslinked peptide candidates, we used recombinant human transglutaminase to label susceptible glutamines with dansyl cadaverine and susceptible lysines with dansyl-aminohexyl-QQIV in MAP-rich tubulin. This method assumes that the naturally-occurring KQ crosslink does not use 100% of a particular peptide, but leaves a substantial portion free for reactions with transglutaminase. A second assumption is that naturally-occurring KQ crosslinks are produced by transglutaminase. This analysis is bolstered by the fact that transglutaminase reacts only with specific glutamine/lysine residues located on accessible surfaces of proteins. For example only one lysine out of a total of 10 lysines in RNase (P61823) is available for modification by transglutaminase [32]. Only one glutamine out of a total of 18 glutamines in the heavy chain of IgG is available for modification by transglutaminase [33]. The expectation is that transglutaminase will label glutamines and lysines that are found in naturally-occurring crosslinks. Mass spectral data of the tryptic peptides from these reactions were checked for the presence of labeled peptides. Peptides modified on glutamine by dansyl cadaverine were identified in MS/MS spectra with the aid of signature fragment ions at 447.2, 402.2, and 336.2 m/z [34]. Peptides modified on lysine by dansyl-aminohexyl QQIV were identified with the aid of signature fragment ions at 475.20, 364.17, 347.14, 234.06, and 170.10 m/z [35]. A dansyl-aminohexyl QQIV adduct on K368 of Tau was found, confirming the availability of this lysine for naturally-occurring crosslinking of the Tau peptide, see Fig 4. No dansyl cadaverine adduct on the crosslink partner Q112 of NFH was found. This is likely due to the very low abundance of NFH in MAP-rich tubulin *Sus scrofa*.

### KQ crosslinked dipeptide prepared from chemically synthesized peptides and transglutaminase

**Fig 5:**
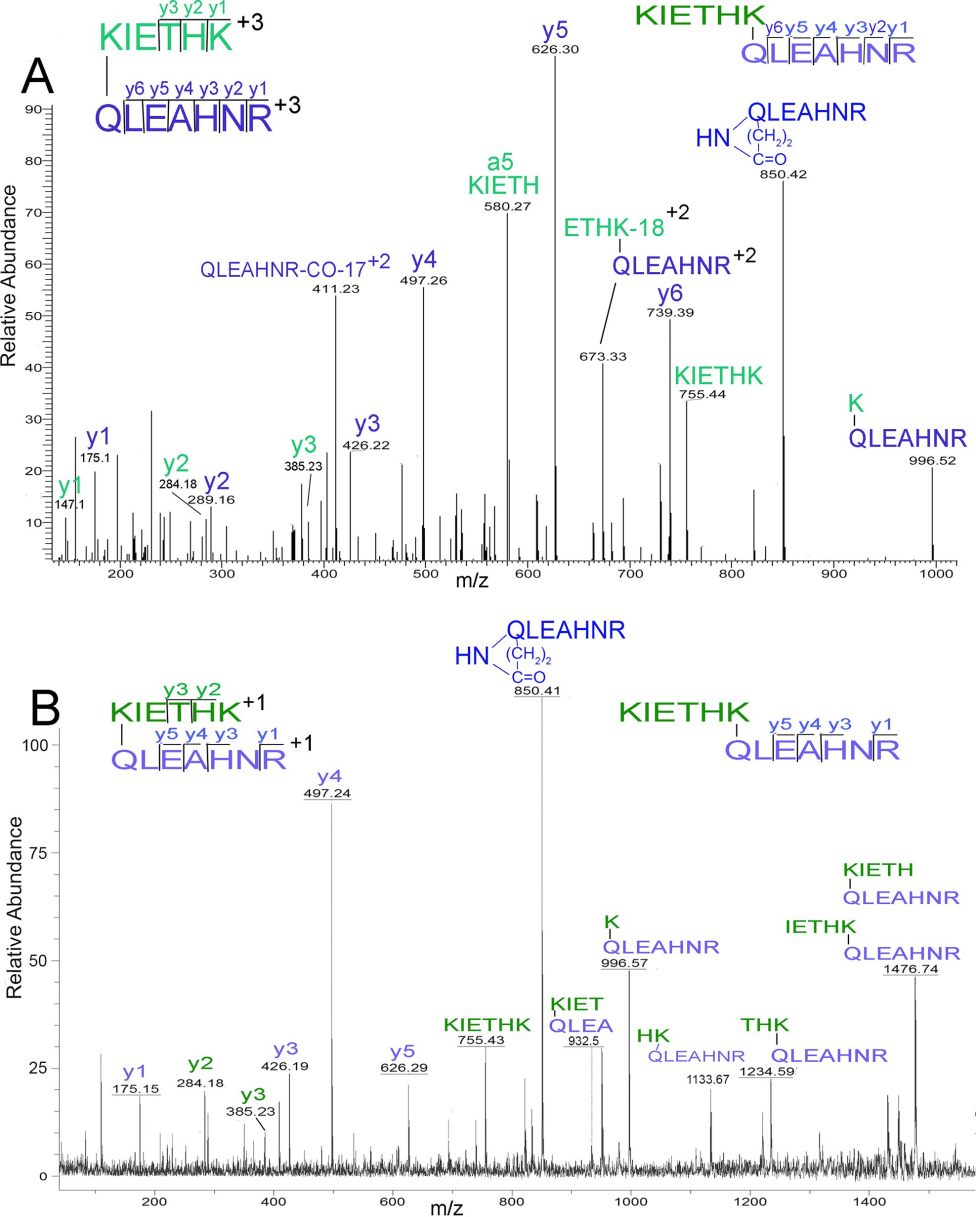
Chemically synthesized peptides crosslinked by bacterial transglutaminase. Panel A is an Orbitrap spectrum of the crosslinked product after tryptic digestion. The triply-charged parent ion has a mass of 535.62 m/z. Panel B is a MALDI-TOF/TOF spectrum of the same peptide. The singly-charged parent ion has a mass of 1604.86 m/z.

We synthesized the crosslinked peptide in Fig 3B to validate our interpretation of that spectrum. This was accomplished by enzymatically linking two chemically synthesized peptides with the aid of bacterial transglutaminase. The MS/MS spectrum acquired on the Orbitrap mass spectrometer is shown in Fig 5A and the spectrum acquired on the Bruker MALDI-TOF/TOF mass spectrometer is shown in Fig 5B.

The parent ion in the Orbitrap spectrum (Fig 5A) is in charge state +3, and in the MALDI-TOF/TOF spectrum (Fig 5B) in charge state +1. Both spectra have ions consistent with crosslinks to either the N-terminal or C-terminal lysine in peptide KIETHK. The green colored y1, y2 and y3 ions in the Orbitrap spectrum Fig 5A support a link to the N-terminal K, while the 673.33 ion supports a link to the C-terminal K. In the MALDI-TOF/TOF spectrum, Fig 5B, the ions that support a link to the N-terminal K are the green colored y2, y3 and the 933.4 ion, while ions that support a link to the C-terminal K are at 1133.67 and 1234.59 m/z. The ion at 1476.74 fits a link to either the N-terminal or C-terminal K. These spectra show that bacterial transglutaminase has the ability to catalyze linkage to K located in the middle of a short peptide as well as K located at the C-terminal of a short peptide.

The MS/MS spectra in Fig 5A and 5B are not identical despite the fact that the same peptide was fragmented in both cases. This is not surprising in view of the fact that the spectra were taken with different mass spectrometers using different fragmentation techniques, and are from parent ions of different charge states. Nevertheless, both spectra define the same crosslinked peptide pair. The Orbitrap spectrum for the model crosslinked peptide in Fig 5A is not identical to the Orbitrap spectrum for the isolated native crosslinked peptide in Fig 3B. This may be a reflection of the different parent ion charge states. However, both spectra share major signals at 850.4, 626.3, 385.2, 289.1, 184.1, 175.1, and 147.1 m/z, all of which are consistent with fragments from the KIETHK/QLEAHNR crosslinked peptide. This correspondence between model and experimental results validates the experimental interpretation.

### Chemically induced KE protein crosslink

**Fig 6:**
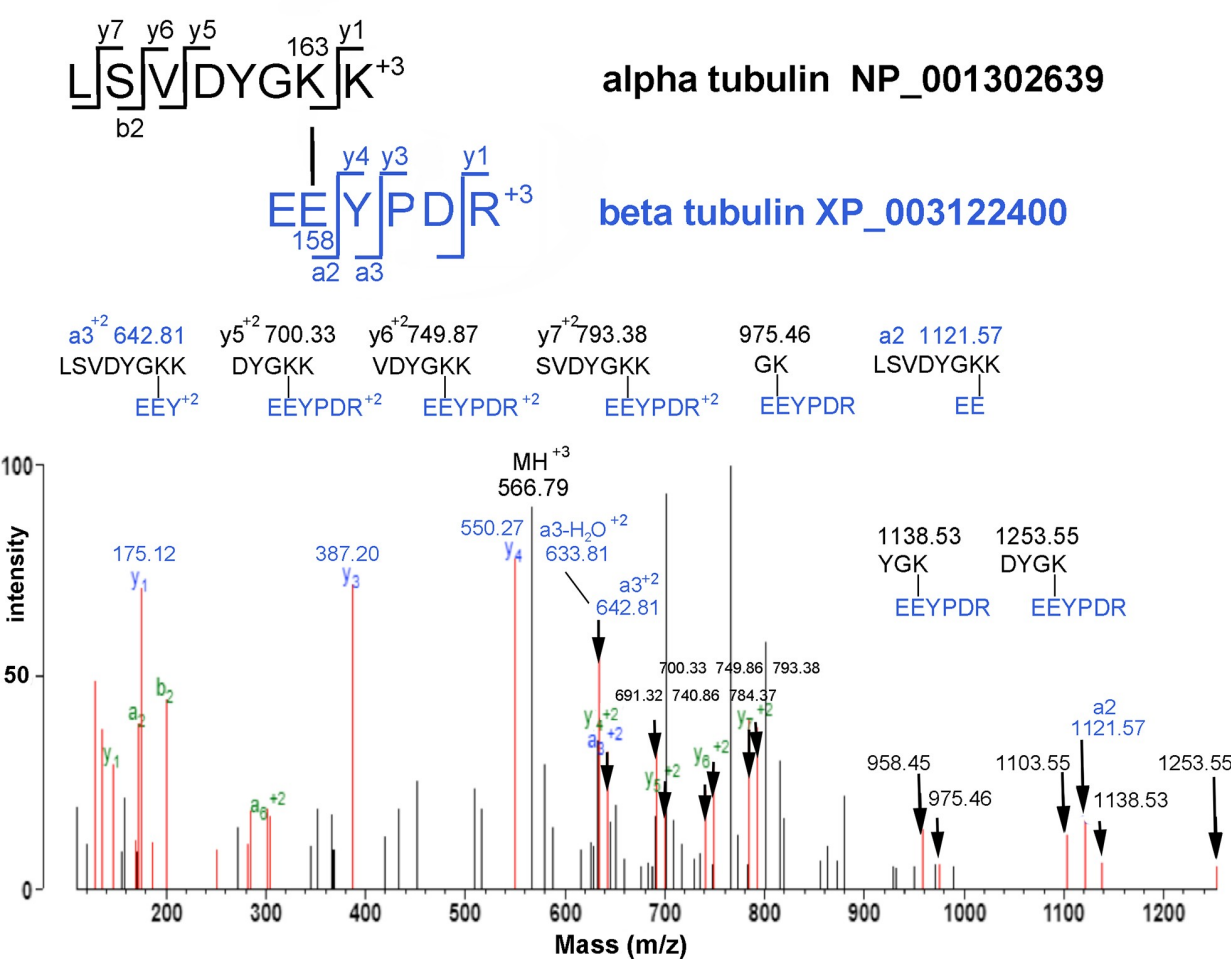
MS/MS spectrum of an isopeptide crosslink between K163 of tubulin alpha-1A and E158 of tubulin beta-4B induced by treatment of MAP-rich tubulin *Sus scrofa* with 100 μM chlorpyrifos oxon. The parent ion at 566.79 is in charge state +3. Arrows point to 14 crosslink-specific fragment ions. Structures are shown for 8 crosslink-specific fragment ions. Fragment ions from both peptides are present. The Protein Prospector score is 29.8 and the score difference is 6.2. The matched intensity is 47.1%. Unassigned, black peaks could not be fit to any sequence.

The search for naturally-occurring KQ crosslinked peptides required the establishment of criteria for accepting or rejecting potential isopeptide crosslink products identified by Protein Prospector. These criteria were applied to our study of KE/KD isopeptide crosslinks induced by exposure of MAP-rich tubulin to 100 μM chlorpyrifos oxon. Fig 6 shows the MS/MS spectrum for K163 from tubulin alpha-1A crosslinked to E158 of tubulin beta-4B as a consequence of incubation with chlorpyrifos oxon.

We believe that the crosslink in Fig 6 is correct based on the following observations. The spectrum includes 14 crosslink-specific ions marked with an arrow. The intensities of all crosslink-specific ions are well above background. A y-ion sequence (YPDR) from one peptide is shown in blue and a b-ion/a-ion sequence (LSV) for the other is shown in green. The Protein Prospector score is 29.8 and the score difference is 6.2. The unassigned intense ions at 702.31, 766.30 and 801.39 did not fit a peptide. The indicated crosslink to E158 could not be distinguished from a crosslink to E157. Crosslinking to D161 could be excluded on the basis of the y3-ion (PDR) at 387.20 m/z. The isopeptide crosslink between K and E is chemically induced and is not a product of transglutaminase activity because MAP-rich tubulin that was not treated with chlorpyrifos oxon did not exhibit this crosslinked peptide.

**Fig 7:**
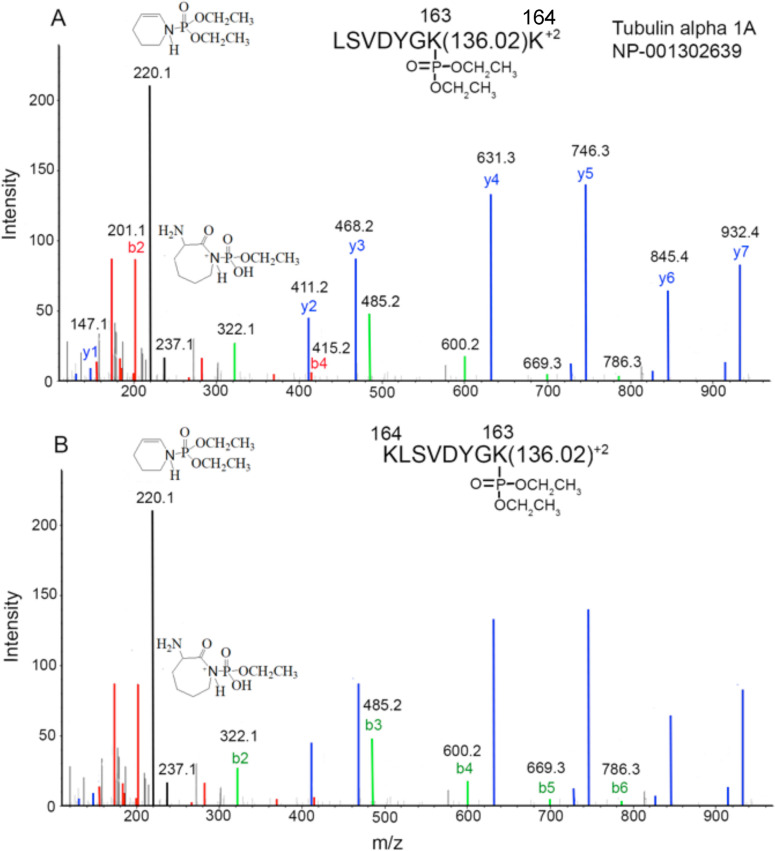
MS/MS spectrum of a diethoxyphospho-adduct on lysine 163 from tubulin alpha-1A. A). Blue masses define a y-ion sequence. Red masses define a b-ion sequence. Unlabeled blue and red lines represent fragments minus NH_3_, H_2_O or CO. The structures at 220.1 and 237.1 m/z represent characteristic ions for diethoxyphospho-lysine. B) Green masses define the b-ion sequence for K(dep)LSVDYGK, the rearranged form of the original peptide.

Additional supporting evidence for the chemically induced crosslink in Fig 6 comes from the fact that a peptide from tubulin alpha-1A with diethoxyphospho-lysine (dep-Lys) at position 163 was present in the peptide mixture that contains the crosslink. Fig 7A shows the MS/MS spectrum for this diethoxyphosphate adduct, LSVDYGK(dep)K. A complete y-ion sequence, including the mass interval for the Lys-dep amino acid at y2 (128 + 136 m/z), is shown in blue (Fig 7A). A partial b-ion sequence is shown, in red. Structures for characteristic ions for diethoxyphospho-lysine are shown at 220.1 and 237.1 m/z [36]. The C-terminal lysine is not the adduct site based on its appearance as y1 in the y-ion sequence.

In addition, there is a rearranged form of the peptide that appears as a b-ion sequence, K(dep)LSVDYGK, shown in green (Fig 7B). Such rearrangements can occur during tryptic digestion. The process requires a peptide containing a missed cleavage site within 2-residues of either terminus. The result is transfer of the amino acid(s) at the missed cleavage site from one terminus to the other, e.g. TITADTFRK to KTITADTFR [37].

**Fig 8:**
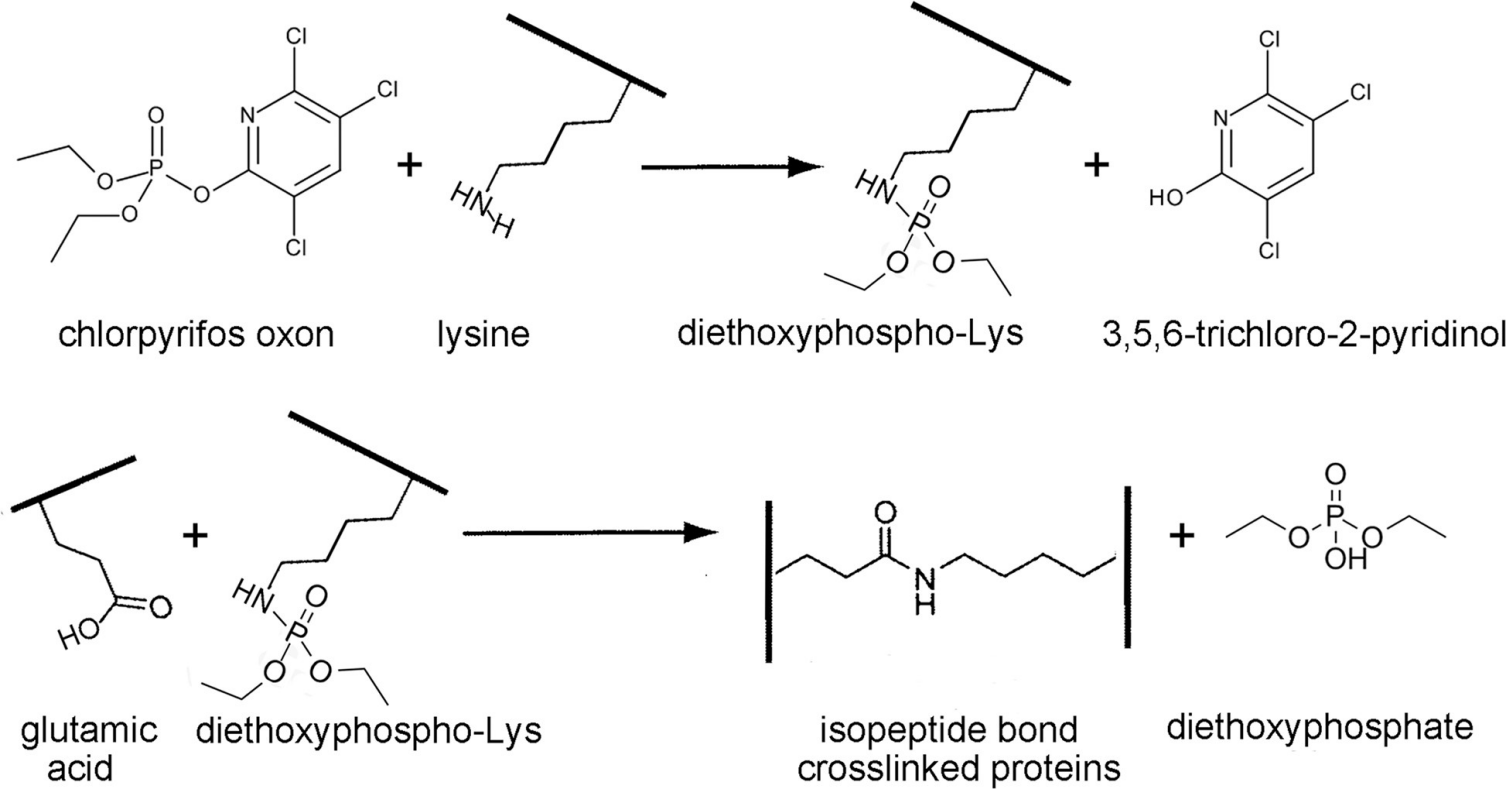
Chemically induced protein crosslinking is initiated when chlorpyrifos oxon makes a covalent bond with lysine to make diethoxyphospho-lysine. The activated diethoxyphospho-lysine reacts with a nearby glutamic acid or aspartic acid to make an isopeptide bond with release of diethoxyphosphate. The KE and KD crosslinks are resistant to trypsin.

Diethoxyphospho-lysine is the activated form of lysine that subsequently reacts with glutamic acid to make the KE crosslink [3, 4]. See Fig 8.

## Discussion

Isopeptide crosslinks between proteins have been implicated in a variety of neurologically important processes including brain function [6, 7] and neuron differentiation[8]. Detrimental effects of isopeptide crosslinking are associated with protein aggregation in senile plaques, neurofibrillary tangles [11], and Lewy bodies [12]. These naturally occurring isopeptide bonds are believed to be the product of transglutaminase activity and would therefore be between lysine and glutamine. We are interested in identifying isopeptide crosslinking in neurological preparations induced by organophosphorus compounds. These crosslinks would be between lysine and glutamic acid or lysine and aspartic acid. In previous studies, we have shown that pure tubulin is particularly receptive to organophosphate induced isopeptide crosslinking [3–5]. To expand upon this observation, we have chosen commercially available porcine MAP-rich tubulin as a more complex model system with which to sort out procedures for identifying relevant isopeptide crosslinks. In the current study, we have endeavored to establish a set of criteria for identifying isopeptide crosslinked peptides in mass spectral MS/MS fragmentation data, and to use those criteria to identify naturally occurring KQ isopeptide bonds and chlorpyrifos oxon induced KE isopeptide bonds in MAP-rich tubulin preparations.

In our search for naturally occurring KQ isopeptides, we separated the proteins in MAP-rich tubulin on SDS gels, prepared tryptic peptides from 29 gel slices taken from 5 SDS gels, and subjected each set of peptides to LC-MS/MS mass spectrometry on a high resolution Orbitrap Fusion Lumos Tribrid mass spectrometer. Three separate preparations were made. This yielded a total of 301,364 MS/MS data files. A Protein Prospector/Batch-Tag Web analysis of these files for KQ isopeptide crosslinked peptides yielded 802 potential crosslinked peptides. We manually screened all 802 MS/MS spectra using our criteria and accepted one. Immunopurification using a commercially available anti-isopeptide monoclonal antibody confirmed the presence of one isopeptide crosslink in MAP-rich tubulin.

### Criteria for accepting software-selected, isopeptide crosslinked peptides

Guidelines are available for evaluating mass spectrometry data for peptides crosslinked by chemicals such as disuccinimidyl suberate and disuccinimidyl glutarate [23, 24, 38]. We have adapted those guidelines for use with isopeptide crosslinks. It is generally accepted that the longer peptide in the crosslinked pair is defined by more fragment ions than the shorter peptide. Absence of fragment ions from the shorter peptide makes a crosslink assignment doubtful, because the assignment relies heavily on the mass of the parent ion. However, the same parent ion mass can belong to both a linear peptide and a crosslinked peptide, making it risky to rely on the parent ion mass without additional evidence for the crosslink. Consequently, one rule for accepting a candidate crosslink is that fragment ions from both peptides must be present in the MS/MS spectrum. A second rule excludes short peptides. Leitner et al. [23] “excluded cross-link identifications that contained a peptide shorter than six amino acids as they were found to contain a disproportionally high number of false positives.” We have chosen to follow the more liberal position of Trnka et al. [24], requiring that peptides in a crosslink candidate contain at least four residues. We justify this position by the fact that we manually examine all the candidate spectra that appear promising.

A third rule requires the presence of crosslink specific ions in the MS/MS spectrum. Crosslink specific ions are defined as ions that include portions of both peptides and the bridge element. Examples of the structures of crosslink specific ions are in Fig 6. We require a minimum of one crosslink specific ion to accept a crosslinked peptide. The greater the number of crosslink specific ions, the better is the confidence of the assignment. Since these diagnostic ions usually have a low intensity, confidence in the assignment is improved when their intensity is at least 5-fold above background. Requiring at least one crosslink specific ion is a conservative rule that could result in discarding valid crosslinked peptides. For example if there are 4 y-ions from each peptide in the spectrum, then both peptides could be taken to be present, with high confidence. That would imply the presence of a crosslink even if there were no crosslink specific ions in the spectrum. But without crosslink specific ions we would discard this candidate.

A fourth rule derives from the observation that peaks that cannot be assigned to the crosslinked peptides are always present in MS/MS spectra. Iacobucci and Sinz (2017) [38] propose that a crosslink product should only be accepted if the majority of fragment ions can be assigned and if the signal to noise ratio is high. The fraction of fragment ions associated with the crosslinked peptide is reflected in the % matched intensity score from Protein Prospector’s Batch-Tag/Search Compare. As an initial criterion, acceptable crosslink assignments must have a % matched intensity score of approximately 40% or greater.

A corollary to the fourth guideline is that the data must be searched to determine whether a linear peptide fits the data better than the crosslink. We performed these analyses in two ways, first by measuring the intervals between peaks (manual sequencing) and second by querying the data with MS-Tag in Protein Prospector.

A fifth rule is that the Protein Prospector score plus the score difference be equal to or greater than 20, and the score difference be greater than zero. However, the scores alone do not predict which candidate crosslink is most convincing.

### Isopeptide crosslink in a linear peptide sequence

**Fig 9:**
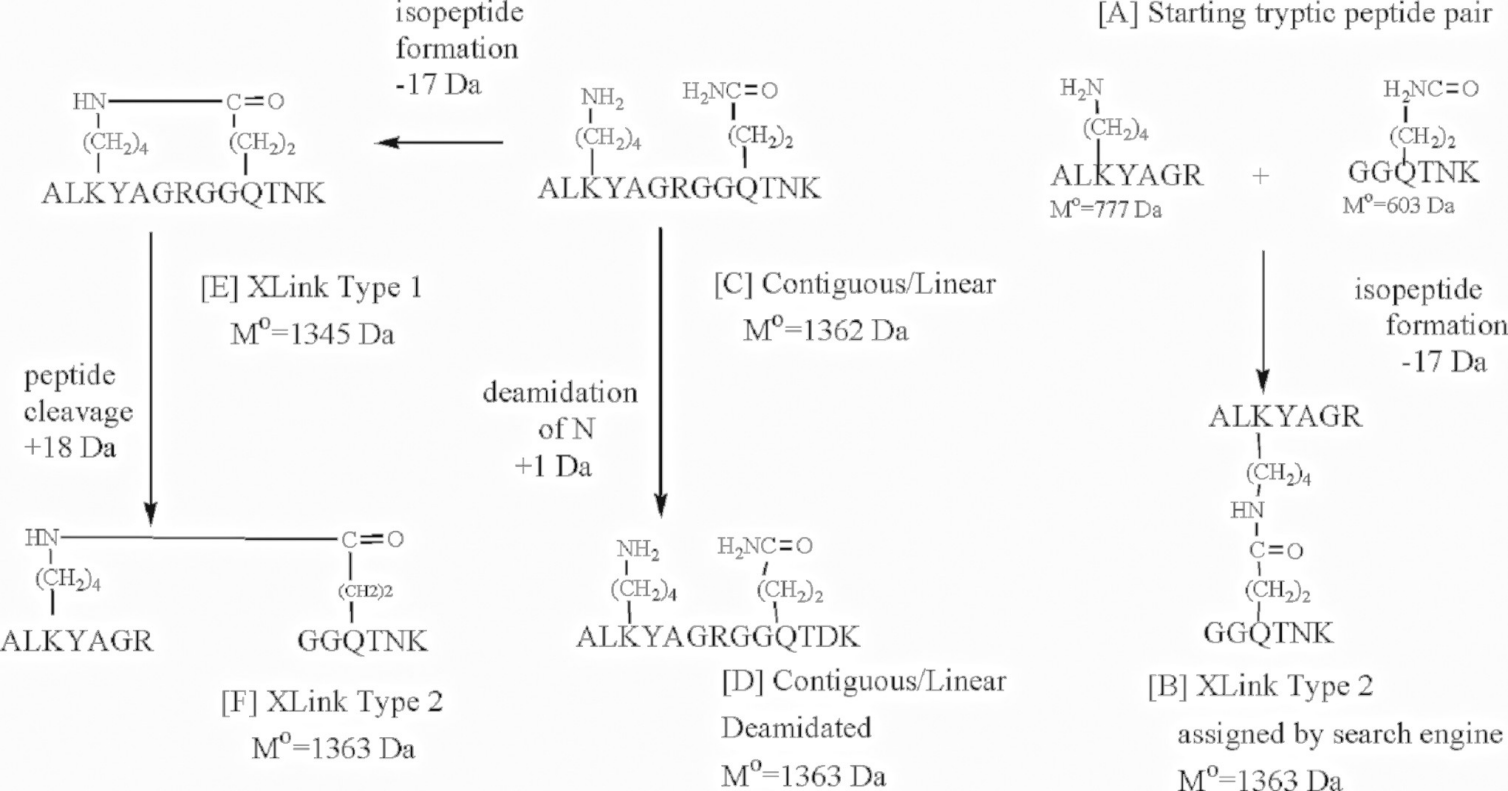
A scheme for possible structures that could be associated with an isopeptide crosslink in a linear peptide sequence.

KQ crosslinks between peptides from the same protein are complicated by the fact that search engines select pairs of peptides to be crosslink candidates without regard to whether both peptides are from the same protein. If the crosslink peptide candidates are from the same protein, they may be contiguous which is a source of complication. To help clarify the discussion on this point we have included Fig 9.

On the right side of Fig 9, there is a representative pair of tryptic peptides (structure A) that might be chosen by a search engine as a candidate for crosslinking. The KQ isopeptide crosslink that a search engine would assign to this peptide pair (a Type 2 crosslink with loss of 17 Da) is shown in structure B. If these peptides are from the same protein, they might be contiguous, forming a linear sequence. The left side of Fig 9 depicts the situation in which the peptides are contiguous. The peptide in structure C shows the peptides from structure A as a contiguous sequence. Formation of structure C from structure A would entail a loss of 18 Da. This contiguous structure would not be flagged by a search algorithm looking for a KQ isopeptide crosslink because formation of a KQ crosslink is associated with a loss of 17 Da. However, if the contiguous peptide underwent deamidation from Asn or Gln, 1 Da would be added to structure C yielding an overall mass loss of 17 Da (structure D shows deamidation of Asn). Structure D is isomeric with structure B, meaning that the linear structure D could be mistaken for an isopeptide crosslinked peptide. In addition, the mass spectrometer can occasionally introduce 1 Da into a mass. This also could make the linear peptide in structure C appear to be an isopeptide crosslink with loss of 17 Da.

The linear peptide in structure C also could undergo an internal isopeptide crosslinking (Type 1 crosslink) that would lead to structure E, with a net loss of 35 Da from the starting peptide pair (structure A). Structure E would not be flagged as a KQ isopeptide crosslink between the original peptide pair because of the 35 Da mass loss associated with its formation. However, if the main peptide chain in structure E were hydrolyzed at a site between K and Q (for example by a tryptic cleavage at Arg with addition of 18 Da) then a Type 1 isopeptide crosslinked peptide would be created (structure F) with a net loss of 17 Da relative to structure A. The isopeptide crosslink in structure F is identical to the isopeptide crosslink in structure B (the only difference being the route by which each was formed) and isomeric to the contiguous/linear structure D.

The challenge in this scenario is discriminating between isomers D and F because either might be observed when starting with a contiguous/linear peptide (structure C). Structures D and F might be differentiated by determining N-terminal b-ion and C-terminal y-ion sequences. In the example, an N-terminal b-ion sequence of GG could occur for structure F but not for structure D. An N-terminal b-ion sequence of AL could appear for either. A C-terminal y-ion sequence of RGAY could occur for structure F, but not for structure D. Structure D also might be identified by performing a non-crosslinked database search for deamidation of Gln or Asn. Alternatively, manual sequencing of the crosslinked candidate might reveal a deamidated Gln or Asn in an otherwise linear sequence. If the isopeptide in crosslinked structure F could be identified then the crosslink interpretation would be substantiated.

### The isopeptide bond can break in the mass spectrometer

**Fig 10:**
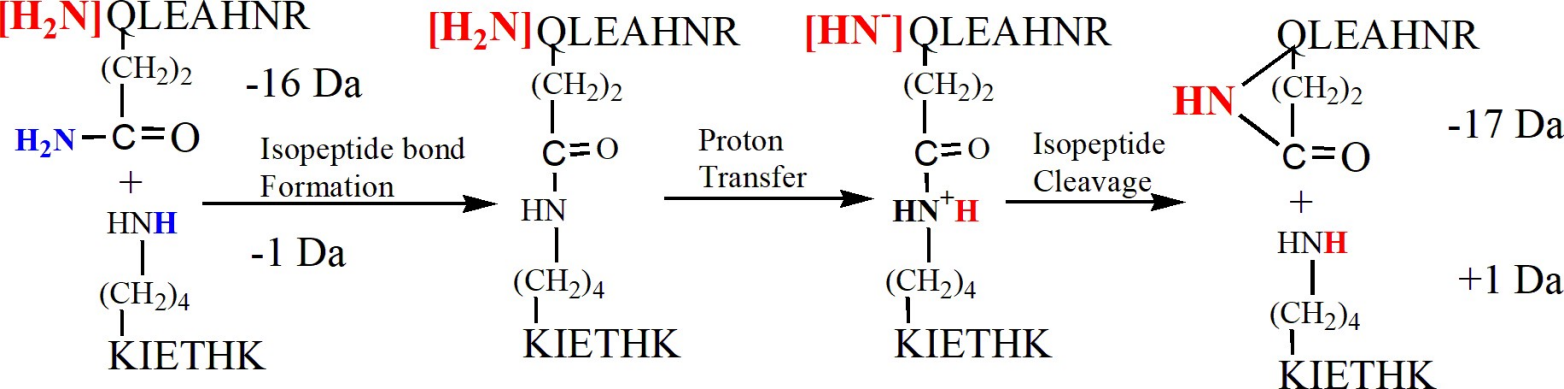
A proposed mechanism for cleavage of the isopeptide bond in the mass spectrometer.

Isopeptide bonds are generally considered to be resistant to fragmentation in the mass spectrometer [39]. However, there are exceptions to this generalization [40]. Peaks consistent with cleavage of the isopeptide bond are present in Figs 3B, 5A and 5B. The isopeptide bond cleavage products are KIETHK (755.43 m/z) and QLEAHNR -17 (850.41 m/z). Cleavage occurred by collision induced dissociation in the Orbitrap and by laser-induced dissociation in the MALDI-TOF/TOF mass spectrometer. The ions at 755.43 m/z and 850.41 m/z provide a basis for proposing a scheme for the fragmentation of the isopeptide bond. The proposed mechanism is in Fig 10.

The first step is formation of the isopeptide bond between peptides QLEAHNR and KIETHK with loss of 16 Da from Gln in peptide QLEAHNR and loss of 1 Da from Lys in peptide KIETHK. The QLEAHNR peptide is shown with the N-terminal amine highlighted in red. The NH_2_ portion of the amide group from Gln and one H from the amine group of Lys are destined to be removed upon formation of the isopeptide bond. These residues are highlighted in blue. The second step proposes a proton transfer from the N-terminal of the QLEAHR peptide to the amide nitrogen in the isopeptide linkage. Such a protonation would activate the isopeptide bond for cleavage in much the same way that protonation of a peptide amide in the main chain activates that bond for cleavage [41]. Isopeptide bond cleavage would be promoted by attack of the N-terminal nitrogen from the QLEAHNR peptide on the carbonyl carbon of the isopeptide bond. The result is a QLEAHNR -17 fragment at 850.41 m/z, where the -17 Da is a combination of the

-16 Da lost during isopeptide bond formation and the 1 Da transferred to the KIETHK peptide during isopeptide bond fragmentation. In addition, the intact KIETHK peptide at 755.43 m/z is a product of cleavage of the isopeptide bond. It is worth repeating that the 850.41 and 755.43 m/z masses appear in the Orbitrap electrospray ionization spectra, 5A and 3B, and the MALDI spectrum 5B. This demonstrates that the proposed fragmentation mechanism is not restricted to a single type of fragmentation process.

Support for our proposed mechanism comes from S1 Fig in the paper by Kang and Baker [42]. This figure shows fragments from the isopeptide crosslinked peptide pair LTVTKNL and NSL that are consistent with cleavage of the isopeptide bond via the mechanism in Fig 10. A fragment for the asparagine containing peptide NSL minus 17 Da is present at 316.14 m/z, as is a fragment for the intact mass of the lysine containing peptide LTVTKNL at 788.49 m/z. As in our example, a proton appears to have been transferred from the peptide containing the amido portion of the isopeptide bond to the peptide containing the Lys portion.

### Chemically induced isopeptide crosslinks

Isopeptide crosslinked peptides lose 17 Da with release of ammonia when the bond is the KQ product of transglutaminase activity, or they lose 18 Da with release of water when the KE or KD bond is induced by chlorpyrifos oxon [3, 4]. Because mass spectrometry identifies the peptide fragments involved in the isopeptide crosslink, it is possible to distinguish between transglutaminase and chemically induced isopeptide crosslinks.

Examination of the KE/KD isopeptide crosslinked peptide pair gives no clue to the chemical that induced the crosslink, because no trace of the chemical remains in the isopeptide link (see Fig 8). However, a precursor of the isopeptide link having a chemical adduct on the crosslinked lysine may be present in the sample along with the crosslink. For example when a diethoxyphospho-lysine adduct is present, it identifies the initiating chemical as an organophosphorus pesticide, such as chlorpyrifos oxon.

The initial screening criteria for accepting isopeptide crosslinks produced by the action of transglutaminase are applicable to isopeptide crosslinks produced by the action of chemicals. It will be important to use these criteria in future studies that aim to understand why exposure to pesticides is associated with increased risk of neurodegenerative disease [27, 43, 44].

## Conclusion

An isopeptide KQ crosslink was found in a commercial MAP-rich tubulin preparation. This indicates that a naturally-occurring KQ crosslink is present in MAP-rich tubulin, suggesting a functional role for this crosslink in-vivo, and implying that transglutaminase participates in its formation. Mass spectrometry is a critical tool in the elucidation of these crosslinks. The criteria we have suggested in the current work should aid in the use of mass spectrometry for identifying isopeptide crosslinks in future studies.

Mass spectrometry also can be used to study organophosphate-induced neurodegenerative disease. To date we have established that pure proteins treated with OP form high molecular weight aggregates and that the aggregated proteins are crosslinked through isopeptide bonds [3, 4]. Evidence from in vivo studies is not yet available, but will benefit from having a set of rules for accepting and rejecting potential crosslinks.

## Supporting information

S1 Table Proteins in MAP-rich tubulin *Sus Scrofa*. (DOCX)

S2 Table Mass differences for dehydro-amino acids in peptide sequences. (DOCX)

S1 Fig Screen shot of Batch-Tag-Web page in protein prospector. (DOCX)

S2 Fig False positive crosslink. (DOCX)

S3 Fig Manual evaluation of +3 and +2 ions. (DOCX)

S4 Fig MSMS of isopeptide in MAP-rich tubulin. (DOCX)

S1 Text Manual evaluation protocol. (DOCX)

S1 Raw image (DOCX)

S1 Graphic (TIF)

## Abbreviations

BLAST: Basic Local Alignment Search Tool from NCBI
dep: diethoxyphosphate
LC-MS/MS: liquid chromatography tandem mass spectrometry
MALDI-TOF/TOF: matrix assisted laser desorption-ionization time of flight/time of flight tandem mass spectrometer
MAP: microtubule associated proteins
MS/MS spectrum: fragmentation mass spectrum
MWCO: molecular weight cut off
NCBI: National Center for Biotechnology Information
NFH: neurofilament heavy polypeptide
OP: organophosphorus toxicants
PBS: phosphate buffered saline
PSM: peptide spectral match
SDS: sodium dodecyl sulfate
TG: transglutaminase

## References

[1] Lorand L, Iismaa SE. Transglutaminase diseases: from biochemistry to the bedside. FASEB J. 2019;33(1):3–12. doi: 10.1096/fj.201801544R 30593123

[2] Folk JE, Finlayson JS. The epsilon-(gamma-glutamyl)lysine crosslink and the catalytic role of transglutaminases. Advances in protein chemistry. 1977;31:1–133. doi: 10.1016/s0065-3233(08)60217-x 73346

[3] Schopfer LM, Lockridge O. Mass Spectrometry Identifies Isopeptide Cross-Links Promoted by Diethylphosphorylated Lysine in Proteins Treated with Chlorpyrifos Oxon. Chem Res Toxicol. 2019;32(4):762–72. doi: 10.1021/acs.chemrestox.9b00001 30844252

[4] Schopfer LM, Lockridge O. Chlorpyrifos oxon promotes tubulin aggregation via isopeptide cross-linking between diethoxyphospho-Lys and Glu or Asp: Implications for neurotoxicity. The Journal of biological chemistry. 2018;293(35):13566–77. doi: 10.1074/jbc.RA118.004172 30006344 PMC6120212

[5] Schmidt C, Breyer F, Blum MM, Thiermann H, Worek F, John H. V-type nerve agents phosphonylate ubiquitin at biologically relevant lysine residues and induce intramolecular cyclization by an isopeptide bond. Anal Bioanal Chem. 2014;406:5171–85. doi: 10.1007/s00216-014-7706-y 24652148

[6] Bailey CD, Johnson GV. Developmental regulation of tissue transglutaminase in the mouse forebrain. J Neurochem. 2004;91(6):1369–79. doi: 10.1111/j.1471-4159.2004.02825.x 15584913

[7] Algarni AS, Hargreaves AJ, Dickenson JM. Activation of transglutaminase 2 by nerve growth factor in differentiating neuroblastoma cells: A role in cell survival and neurite outgrowth. Eur J Pharmacol. 2018;820:113–29. doi: 10.1016/j.ejphar.2017.12.023 29242118

[8] Tucholski J, Lesort M, Johnson GV. Tissue transglutaminase is essential for neurite outgrowth in human neuroblastoma SH-SY5Y cells. Neuroscience. 2001;102(2):481–91. doi: 10.1016/s0306-4522(00)00482-6 11166134

[9] Lorand L. Crosslinks in blood: transglutaminase and beyond. FASEB J. 2007;21(8):1627–32. doi: 10.1096/fj.07-0602ufm 17538029

[10] Hitomi K. Transglutaminases in skin epidermis. European journal of dermatology: EJD. 2005;15(5):313–9. 16172037

[11] Wilhelmus MM, Grunberg SC, Bol JG, van Dam AM, Hoozemans JJ, Rozemuller AJ, et al. Transglutaminases and transglutaminase-catalyzed cross-links colocalize with the pathological lesions in Alzheimer’s disease brain. Brain Pathol. 2009;19(4):612–22. doi: 10.1111/j.1750-3639.2008.00197.x 18673368 PMC8094859

[12] Nemes Z, Petrovski G, Aerts M, Sergeant K, Devreese B, Fesus L. Transglutaminase-mediated intramolecular cross-linking of membrane-bound alpha-synuclein promotes amyloid formation in Lewy bodies. The Journal of biological chemistry. 2009;284(40):27252–64. doi: 10.1074/jbc.M109.033969 19651786 PMC2785653

[13] Lorand L, Hsu LK, Siefring GE Jr., Rafferty NS. Lens transglutaminase and cataract formation. Proc Natl Acad Sci U S A. 1981;78(3):1356–60. doi: 10.1073/pnas.78.3.1356 6112745 PMC319129

[14] Lexhaller B, Ludwig C, Scherf KA. Identification of Isopeptides Between Human Tissue Transglutaminase and Wheat, Rye, and Barley Gluten Peptides. Scientific reports. 2020;10(1):7426. doi: 10.1038/s41598-020-64143-9 32367038 PMC7198585

[15] Murthy SN, Wilson JH, Lukas TJ, Kuret J, Lorand L. Cross-linking sites of the human tau protein, probed by reactions with human transglutaminase. J Neurochem. 1998;71(6):2607–14. doi: 10.1046/j.1471-4159.1998.71062607.x 9832162

[16] Zhang J, Wang S, Huang W, Bennett DA, Dickson DW, Wang D, et al. Tissue Transglutaminase and Its Product Isopeptide Are Increased in Alzheimer’s Disease and APPswe/PS1dE9 Double Transgenic Mice Brains. Mol Neurobiol. 2016;53(8):5066–78. doi: 10.1007/s12035-015-9413-x 26386840 PMC4799778

[17] Johnson GV, LeShoure R Jr. Immunoblot analysis reveals that isopeptide antibodies do not specifically recognize the epsilon-(gamma-glutamyl)lysine bonds formed by transglutaminase activity. Journal of neuroscience methods. 2004;134(2):151–8. doi: 10.1016/j.jneumeth.2003.11.006 15003381

[18] El-Hofi M, Ismail A, Nour M, Ibrahim O. Isolation, purification and characterisation of transglutaminase from rosemary (Rosmarinus officinalis L.) leaves. Acta scientiarum polonorum Technologia alimentaria. 2014;13(3):267–78. doi: 10.17306/j.afs.2014.3.5 24887942

[19] Recktenwald CV, Hansson GC. The Reduction-insensitive Bonds of the MUC2 Mucin Are Isopeptide Bonds. The Journal of biological chemistry. 2016;291(26):13580–90. doi: 10.1074/jbc.M116.726406 27129250 PMC4919444

[20] Lexhaller B, Ludwig C, Scherf KA. Comprehensive Detection of Isopeptides between Human Tissue Transglutaminase and Gluten Peptides. Nutrients. 2019;11(10):2263. doi: 10.3390/nu11102263 31547042 PMC6835481

[21] Schmitt LR, Henderson R, Barrett A, Darula Z, Issaian A, D’Alessandro A, et al. Mass spectrometry-based molecular mapping of native FXIIIa cross-links in insoluble fibrin clots. J Biol Chem. 2019;294:8773–8. doi: 10.1074/jbc.AC119.007981 31028172 PMC6552431

[22] Arike L, Hansson GC, Recktenwald CV. Identifying transglutaminase reaction products via mass spectrometry as exemplified by the MUC2 mucin- pitfalls and traps. Anal Biochem. 2020;597:113668. doi: 10.1016/j.ab.2020.113668 32222540 PMC7184670

[23] Leitner A, Reischl R, Walzthoeni T, Herzog F, Bohn S, Forster F, et al. Expanding the chemical cross-linking toolbox by the use of multiple proteases and enrichment by size exclusion chromatography. Mol Cell Proteomics. 2012;11(3):M111.014126. doi: 10.1074/mcp.M111.014126 22286754 PMC3316732

[24] Trnka MJ, Baker PR, Robinson PJ, Burlingame AL, Chalkley RJ. Matching cross-linked peptide spectra: only as good as the worse identification. Mol Cell Proteomics. 2014;13(2):420–34. 10.1074/mcp.M113.034009 PMC3916644 24335475

[25] Rinner O, Seebacher J, Walzthoeni T, Mueller LN, Beck M, Schmidt A, et al. Identification of cross-linked peptides from large sequence databases. Nat Methods. 2008;5:315–8. doi: 10.1038/nmeth.1192 18327264 PMC2719781

[26] Lenz S, Giese SH, Fischer L, Rappsilber J. In-search assignment of monoisotopic peaks improves the identification of cross-linked peptides. J Proteome Res. 2018;17:3923–31. doi: 10.1021/acs.jproteome.8b00600 30293428 PMC6279313

[27] Hayden KM, Norton MC, Darcey D, Ostbye T, Zandi PP, Breitner JC, et al. Occupational exposure to pesticides increases the risk of incident AD: the Cache County study. Neurology. 2010;74(19):1524–30. doi: 10.1212/WNL.0b013e3181dd4423 20458069 PMC2875926

[28] Peeples ES, Schopfer LM, Duysen EG, Spaulding R, Voelker T, Thompson CM, et al. Albumin, a new biomarker of organophosphorus toxicant exposure, identified by mass spectrometry. Toxicol Sci. 2005;83(2):303–12. doi: 10.1093/toxsci/kfi023 15525694

[29] Sporty JL, Lemire SW, Jakubowski EM, Renner JA, Evans RA, Williams RF, et al. Immunomagnetic separation and quantification of butyrylcholinesterase nerve agent adducts in human serum. Anal Chem. 2010;82(15):6593–600. doi: 10.1021/ac101024z 20617824

[30] Burke MC, Oei MS, M/K/E, Ostrand-Rosenberg S, Fenselau C. Ubiquinate proteins in exosomes secreted by myeloid-derived suppressor cells. J Proteome Res. 2014;13:5965–72. doi: 10.1021/pr500854x 25285581 PMC4261954

[31] Chen M, Zhang M, Zhai L, Hu H, Liu P, Tan M. Tryptic Peptides Bearing C-Terminal Dimethyllysine Need to Be Considered during the Analysis of Lysine Dimethylation in Proteomic Study. J Proteome Res. 2017;16:3460–9. doi: 10.1021/acs.jproteome.7b00373 28730820

[32] Murthy SN, Lukas TJ, Jardetzky TS, Lorand L. Selectivity in the post-translational, transglutaminase-dependent acylation of lysine residues. Biochemistry. 2009;48(12):2654–60. doi: 10.1021/bi802323z 19222223

[33] Jeger S, Zimmermann K, Blanc A, Grunberg J, Honer M, Hunziker P, et al. Site-specific and stoichiometric modification of antibodies by bacterial transglutaminase. Angew Chem Int Ed Engl. 2010;49(51):9995–7. doi: 10.1002/anie.201004243 21110357

[34] Biberoglu K, Schopfer LM, Tacal O, Lockridge O. Characteristic fragment ions associated with dansyl cadaverine and biotin cadaverine adducts on glutamine. Anal Biochem. 2020;600:113718. doi: 10.1016/j.ab.2020.113718 32335065 PMC7302536

[35] Schopfer LM, Lockridge O. Signature Ions in MS/MS Spectra for Dansyl-Aminohexyl-QQIV Adducts on Lysine. Molecules. 2020;25(11):2659. doi: 10.3390/molecules25112659 32521655 PMC7321351

[36] Grigoryan H, Li B, Xue W, Grigoryan M, Schopfer LM, Lockridge O. Mass spectral characterization of organophosphate-labeled lysine in peptides. Anal Biochem. 2009;394(1):92–100 doi: 10.1016/j.ab.2009.07.008 19596251 PMC2735595

[37] Fodor S, Zhang Z. Rearrangement of terminal amino acid residues in peptides by protease-catalyzed intramolecular transpeptidation. Anal Biochem. 2006;356:282–90. doi: 10.1016/j.ab.2006.06.023 16859627

[38] Iacobucci C, Sinz A. To Be or Not to Be? Five Guidelines to Avoid Misassignments in Cross-Linking/Mass Spectrometry. Anal Chem. 2017;89(15):7832–5. doi: 10.1021/acs.analchem.7b02316 28723100

[39] Nemes Z, Devreese B, Steinert PM, Van Beeumen J, Fesus L. Cross-linking of ubiquitin, HSP27, parkin, and alpha-synuclein by gamma-glutamyl-epsilon-lysine bonds in Alzheimer’s neurofibrillary tangles. FASEB J. 2004;18(10):1135–7. doi: 10.1096/fj.04-1493fje 15132984

[40] Kang HJ, Coulibaly F, Clow F, Proft T, Baker EN. Stabilizing isopeptide bonds revealed in gram-positive bacterial pilus structure. Science. 2007;318(5856):1625–8. doi: 10.1126/science.1145806 18063798

[41] Schlosser A, Lehmann WD. Five-membered ring formation in unimolecular reactions of peptides: a key structural element controlling low-energy collision-induced dissociation of peptides. Journal of mass spectrometry: JMS. 2000;35(12):1382–90. doi: 10.1002/1096-9888(200012)35:12&lt;1382::AID-JMS84&gt;3.0.CO;2-6 11180628

[42] Kang HJ, Baker EN. Intramolecular isopeptide bonds: protein crosslinks built for stress? Trends Biochem Sci. 2011;36(4):229–37. doi: 10.1016/j.tibs.2010.09.007 21055949

[43] Jokanovic M. Neurotoxic effects of organophosphorus pesticides and possible association with neurodegenerative diseases in man: A review. Toxicology. 2018;410:125–31. doi: 10.1016/j.tox.2018.09.009 30266654

[44] Kamel F, Hoppin JA. Association of pesticide exposure with neurologic dysfunction and disease. Environ Health Perspect. 2004;112(9):950–8. doi: 10.1289/ehp.7135 15198914 PMC1247187

