# Supplementary material for: Evaluation of mass spectrometry MS/MS spectra for the presence of isopeptide crosslinked peptides
Source: PLoS One. 2021 Jul 9;16(7):e0254450. doi: 10.1371/journal.pone.0254450 (PMC8270460; doi:10.1371/journal.pone.0254450)
Supplement: S1 Table — (DOCX) [file pone.0254450.s001.docx]

PLOS One

Evaluation of mass spectrometry MS/MS spectra for the presence of isopeptide crosslinked peptides

Lawrence M. Schopfer, Seda Onder, Oksana Lockridge

Eppley Institute, University of Nebraska Medical Center, Omaha, NE 68198 USA

Department of Biochemistry, School of Pharmacy, Hacettepe University, Ankara 06100, Turkey

S1 Table. Proteins in MAP-rich tubulin *Sus scrofa*. 6 user proteins are in bold font.

| Accession | Description | # PSM | MW [kDa] |
| --- | --- | --- | --- |
| **NP_001302639.1** | **tubulin alpha-1A chain [Sus scrofa]** | **1115** | **50.1** |
| XP_001928233.2 | LOW QUALITY PROTEIN: tubulin alpha-1D chain [Sus scrofa] | 957 | 50.3 |
| XP_020929709.1 | tubulin alpha-3 chain isoform X1 [Sus scrofa] | 926 | 52.2 |
| XP_001928370.3 | tubulin alpha-4A chain [Sus scrofa] | 857 | 49.9 |
| XP_020955779.1 | tubulin beta-2A chain [Sus scrofa] | 805 | 49.9 |
| NP_001230363.1 | tubulin beta-2B chain [Sus scrofa] | 746 | 49.9 |
| **XP_003122400.2** | **tubulin beta-4B chain [Sus scrofa]** | **685** | **49.8** |
| XP_003480860.1 | tubulin beta-4A chain [Sus scrofa] | 643 | 49.6 |
| NP_001038077.1 | tubulin beta chain [Sus scrofa] | 583 | 49.6 |
| XP_020951690.1 | LOW QUALITY PROTEIN: tubulin beta-6 chain [Sus scrofa] | 350 | 49.8 |
| **XP_013839898.1** | **microtubule-associated protein 2 isoform X8 [Sus scrofa]** | **315** | **213.9** |
| XP_005672205.1 | microtubule-associated protein 2 isoform X2 [Sus scrofa] | 310 | 217.1 |
| XP_020952325.1 | microtubule-associated protein 1A isoform X2 [Sus scrofa] | 173 | 303.2 |
| XP_005659735.3 | microtubule-associated protein 1A isoform X1 [Sus scrofa] | 149 | 328.8 |
| NP_001107168.1 | tubulin beta-1 chain [Sus scrofa] | 106 | 49.5 |
| **XP_003134080.1** | **microtubule-associated protein 1B isoform X1 [Sus scrofa]** | **96** | **270.5** |
| XP_005652632.1 | ATP synthase F(0) complex subunit C2, mitochondrial isoform X1 [Sus scrofa] | 45 | 23.5 |
| NP_001230836.1 | heat shock cognate 71 kDa protein [Sus scrofa] | 36 | 70.8 |
| XP_020922469.1 | microtubule-associated protein tau isoform X12 [Sus scrofa] | 32 | 50.9 |
| **XP_020922473.1** | **microtubule-associated protein tau isoform X16 [Sus scrofa]** | **26** | **45.5** |
| XP_020937243.1 | LOW QUALITY PROTEIN: cytoplasmic dynein 1 heavy chain 1 [Sus scrofa] | 23 | 531.8 |
| XP_001929445.4 | ATP synthase subunit beta, mitochondrial [Sus scrofa] | 20 | 59.9 |
| NP_001138313.1 | hemoglobin subunit beta [Sus scrofa] | 16 | 16.2 |
| XP_013837068.1 | microtubule-associated protein 4 isoform X6 [Sus scrofa] | 14 | 236.9 |
| XP_003124328.1 | actin, cytoplasmic 1 [Sus scrofa] | 12 | 41.7 |
| XP_020941998.1 | CAP-Gly domain-containing linker protein 2 isoform X1 [Sus scrofa] | 12 | 119.2 |
| XP_020920868.1 | serine/threonine-protein kinase DCLK1 isoform X1 [Sus scrofa] | 11 | 82.2 |
| NP_001163988.1 | actin, alpha cardiac muscle 1 [Sus scrofa] | 11 | 42 |
| XP_020934724.1 | microtubule-associated protein 1S isoform X1 [Sus scrofa] | 10 | 110.5 |
| XP_020926262.1 | microtubule-associated protein 4 isoform X35 [Sus scrofa] | 9 | 103.9 |
| XP_003360037.3 | protein-glutamine gamma-glutamyltransferase 2 [Sus scrofa] | 8 | 77.2 |
| XP_020940891.1 | cytoskeleton-associated protein 5 isoform X1 [Sus scrofa] | 7 | 225.5 |
| NP_999423.2 | cAMP-dependent protein kinase type II-alpha regulatory subunit [Sus scrofa] | 7 | 45.1 |
| XP_020950937.1 | alpha-enolase isoform X1 [Sus scrofa] | 7 | 47.3 |
| XP_003358329.1 | vesicle-associated membrane protein 2 [Sus scrofa] | 7 | 12.6 |
| XP_020957147.1 | tripartite motif-containing protein 2 isoform X1 [Sus scrofa] | 6 | 86.8 |
| XP_001927836.1 | dihydropyrimidinase-related protein 2 isoform X1 [Sus scrofa] | 6 | 73.5 |
| NP_001001546.1 | myelin basic protein [Sus scrofa] | 6 | 18.7 |
| XP_020956318.1 | rho GTPase-activating protein 10 isoform X1 [Sus scrofa] | 6 | 89.6 |
| XP_020958258.1 | cAMP-dependent protein kinase type II-beta regulatory subunit [Sus scrofa] | 5 | 46.2 |
| XP_020930806.1 | CLIP-associating protein 1 isoform X1 [Sus scrofa] | 5 | 178.4 |
| XP_005668766.1 | glial fibrillary acidic protein isoform X1 [Sus scrofa] | 5 | 53.6 |
| NP_001164298.1 | microtubule-associated proteins 1A/1B light chain 3A [Sus scrofa] | 5 | 14.3 |
| XP_003132938.1 | phosphatidylethanolamine-binding protein 1 isoform X1 [Sus scrofa] | 4 | 21 |
| NP_001093402.1 | phosphoglycerate kinase 1 [Sus scrofa] | 4 | 44.5 |
| NP_998931.1 | heat shock 70 kDa protein 1B [Sus scrofa] | 4 | 70.1 |
| NP_999138.1 | heat shock protein HSP 90-alpha [Sus scrofa] | 4 | 84.7 |
| XP_020943008.1 | dynactin subunit 1 isoform X1 [Sus scrofa] | 4 | 141.7 |
| XP_020942626.1 | hemoglobin subunit alpha-like [Sus scrofa] | 3 | 15.2 |
| XP_020952020.1 | cAMP-dependent protein kinase catalytic subunit beta isoform X1 [Sus scrofa] | 3 | 46.2 |
| NP_001231582.1 | serotransferrin precursor [Sus scrofa] | 3 | 78.9 |
| XP_020946773.1 | glyceraldehyde-3-phosphate dehydrogenase isoform X1 [Sus scrofa] | 3 | 37.2 |
| XP_020957128.1 | septin-11 isoform X1 [Sus scrofa] | 3 | 51.4 |
| XP_005662644.1 | echinoderm microtubule-associated protein-like 4 isoform X1 [Sus scrofa] | 3 | 111.7 |
| XP_013840158.1 | brain acid soluble protein 1 [Sus scrofa] | 3 | 23.1 |
| NP_999092.1 | aspartate aminotransferase, cytoplasmic [Sus scrofa] | 3 | 46.4 |
| NP_001230504.1 | creatine kinase B-type [Sus scrofa] | 3 | 42.6 |
| XP_005663334.1 | rho guanine nucleotide exchange factor 2 isoform X1 [Sus scrofa] | 3 | 132.4 |
| NP_999495.1 | glucose-6-phosphate isomerase [Sus scrofa] | 3 | 63.1 |
| XP_013851559.1 | galectin-related protein isoform X1 [Sus scrofa] | 3 | 20.3 |
| XP_001928912.1 | alpha-centractin [Sus scrofa] | 3 | 42.6 |
| XP_003359464.1 | kinesin heavy chain [Sus scrofa] | 3 | 109.3 |
| XP_020955351.1 | echinoderm microtubule-associated protein-like 1 isoform X1 [Sus scrofa] | 3 | 98.6 |
| XP_020951585.1 | MAP7 domain-containing protein 1 isoform X5 [Sus scrofa] | 3 | 94.1 |
| XP_003131508.1 | keratin, type I cytoskeletal 10 isoform X1 [Sus scrofa] | 3 | 62.6 |
| XP_003134838.1 | septin-7 isoform X1 [Sus scrofa] | 3 | 50.6 |
| XP_003123401.1 | cAMP-dependent protein kinase catalytic subunit alpha isoform X1 [Sus scrofa] | 3 | 40.6 |
| XP_020924489.1 | 78 kDa glucose-regulated protein isoform X1 [Sus scrofa] | 3 | 73 |
| XP_005656114.2 | LOW QUALITY PROTEIN: elongation factor 1-alpha, somatic form-like [Sus scrofa] | 3 | 55.3 |
| XP_020923210.1 | 14-3-3 protein epsilon isoform X1 [Sus scrofa] | 3 | 29.2 |
| XP_020918184.1 | LOW QUALITY PROTEIN: microtubule-associated protein 6 [Sus scrofa] | 3 | 83.8 |
| NP_001231362.1 | heat shock protein HSP 90-beta [Sus scrofa] | 3 | 83.2 |
| XP_020932220.1 | kinesin-like protein KIF2A isoform X1 [Sus scrofa] | 3 | 84.1 |
| XP_020923850.1 | ras-related protein Rab-5A isoform X1 [Sus scrofa] | 3 | 23.7 |
| XP_005673018.1 | 14-3-3 protein beta/alpha [Sus scrofa] | 3 | 28.1 |
| XP_020932067.1 | lanC-like protein 1 isoform X1 [Sus scrofa] | 2 | 49 |
| XP_003129998.3 | histone H2AX [Sus scrofa] | 2 | 29.5 |
| XP_003134722.3 | centrosomal protein of 41 kDa isoform X1 [Sus scrofa] | 2 | 41 |
| NP_999039.1 | malate dehydrogenase, cytoplasmic [Sus scrofa] | 2 | 36.4 |
| XP_005670496.2 | neurofilament medium polypeptide isoform X1 [Sus scrofa] | 2 | 103.9 |
| XP_020930255.1 | septin-2 isoform X1 [Sus scrofa] | 2 | 46 |
| NP_001116678.1 | tubulin polymerization-promoting protein [Sus scrofa] | 2 | 24 |
| XP_013853381.2 | kinesin-like protein KIF21A isoform X1 [Sus scrofa] | 2 | 187.5 |
| XP_020957861.1 | glutamine synthetase isoform X1 [Sus scrofa] | 2 | 42.9 |
| XP_005652652.1 | gamma-enolase isoform X1 [Sus scrofa] | 2 | 47.2 |
| XP_013849361.2 | LOW QUALITY PROTEIN: spectrin alpha chain, non-erythrocytic 1 [Sus scrofa] | 2 | 286.2 |
| XP_020943878.1 | spectrin beta chain, non-erythrocytic 1 isoform X1 [Sus scrofa] | 2 | 274.3 |
| NP_998928.1 | ubiquitin carboxyl-terminal hydrolase isozyme L1 [Sus scrofa] | 2 | 24.8 |
| NP_001230248.1 | immunoglobulin lambda-like polypeptide 5 precursor [Sus scrofa] | 2 | 24.1 |
| XP_013841444.2 | LOW QUALITY PROTEIN: MAP7 domain-containing protein 2 [Sus scrofa] | 2 | 85.5 |
| NP_999415.1 | platelet-activating factor acetylhydrolase IB subunit alpha [Sus scrofa] | 2 | 46.6 |
| NP_001020400.1 | fatty acid-binding protein, brain [Sus scrofa] | 2 | 14.9 |
| XP_020918452.1 | neural cell adhesion molecule 1 isoform X1 [Sus scrofa] | 2 | 123.6 |
| XP_003353540.1 | actin-related protein 10 [Sus scrofa] | 2 | 46.2 |
| XP_003123268.1 | intercellular adhesion molecule 5 isoform X1 [Sus scrofa] | 2 | 96.5 |
| XP_003131468.2 | 2',3'-cyclic-nucleotide 3'-phosphodiesterase isoform X1 [Sus scrofa] | 2 | 47.2 |
| XP_003483583.1 | phosphoglycerate mutase 1 [Sus scrofa] | 2 | 28.8 |
| XP_020950417.1 | AP-2 complex subunit alpha-1 isoform X1 [Sus scrofa] | 2 | 107.7 |
| XP_013846182.1 | cyclin-dependent-like kinase 5 isoform X1 [Sus scrofa] | 2 | 34.1 |
| XP_020955585.1 | A-kinase anchor protein 5 [Sus scrofa] | 2 | 47.2 |
| NP_001004034.1 | high mobility group protein B1 [Sus scrofa] | 2 | 24.9 |
| XP_020954765.1 | poly [ADP-ribose] polymerase 6 isoform X1 [Sus scrofa] | 2 | 126.3 |
| NP_001005208.1 | serum albumin precursor [Sus scrofa] | 2 | 69.6 |
| XP_003481635.2 | keratin, type II cytoskeletal 1 [Sus scrofa] | 2 | 64.8 |
| XP_003131697.1 | septin-4 isoform X1 [Sus scrofa] | 2 | 55.1 |
| NP_001156363.1 | trypsinogen precursor [Sus scrofa] | 2 | 25.9 |
| XP_020921706.1 | clathrin heavy chain 1 isoform X1 [Sus scrofa] | 2 | 192.3 |
| XP_005664048.1 | nucleosome assembly protein 1-like 1 isoform X1 [Sus scrofa] | 2 | 45.3 |
| **XP_005670835.2** | **LOW QUALITY PROTEIN: neurofilament heavy polypeptide** | **2** | **117.9** |
| XP_020929058.1 | heat shock 70 kDa protein 12A isoform X3 [Sus scrofa] | 2 | 75.9 |
| NP_001230549.1 | cytoplasmic dynein 1 light intermediate chain 2 [Sus scrofa] | 2 | 54.1 |
