# Supplementary material for: Evaluation of mass spectrometry MS/MS spectra for the presence of isopeptide crosslinked peptides
Source: PLoS One. 2021 Jul 9;16(7):e0254450. doi: 10.1371/journal.pone.0254450 (PMC8270460; doi:10.1371/journal.pone.0254450)
Supplement: S2 Table — (DOCX) [file pone.0254450.s002.docx]

Evaluation of mass spectrometry MS/MS spectra for the presence of isopeptide crosslinked peptides

Lawrence M. Schopfer, Seda Onder, Oksana Lockridge

Eppley Institute, University of Nebraska Medical Center, Omaha, NE 68198 USA

Department of Biochemistry, School of Pharmacy, Hacettepe University, Ankara 06100, Turkey

S2 Table. Mass differences for dehydro-amino acids in peptide sequences

| Amino acid | +1 charge state | +2 charge state | +3 charge state |
| --- | --- | --- | --- |
| Glycine | ∆G 57.0215 | ∆G 28.510 | ∆G 19.0072 |
| Alanine | ∆A 71.0371 | ∆A 35.518 | ∆A 23.6790 |
| Serine | ∆S 87.0320 | ∆S 43.516 | ∆S 29.0107 |
| Proline | ∆P 97.0528 | ∆P 48.527 | ∆P 32.3509 |
| Valine | ∆V 99.0684 | ∆V 49.534 | ∆V 33.0228 |
| Threonine | ∆T 101.0476 | ∆T 50.523 | ∆T 33.6826 |
| Cysteine | ∆C 103.0092 | ∆C 51.504 | ∆C 34.3364 |
| Isoleucine | ∆I 113.0841 | ∆I 56.542 | ∆I 37.6947 |
| Leucine | ∆L 113.0841 | ∆L 56.542 | ∆L 37.6947 |
| Asparagine | ∆N 114.0429 | ∆N 57.021 | ∆N 38.0143 |
| Aspartic acid | ∆D 115.0269 | ∆D 57.513 | ∆D 38.3423 |
| Glutamine | ∆Q 128.0586 | ∆Q 64.029 | ∆Q 42.6862 |
| Lysine | ∆K 128.0950 | ∆K 64.047 | ∆K 42.6983 |
| Glutamic acid | ∆E 129.0426 | ∆E 64.521 | ∆E 43.0142 |
| Methionine | ∆M 131.0404 | ∆M 65.520 | ∆M 43.6802 |
| Histidine | ∆H 137.0590 | ∆H 68.529 | ∆H 45.6863 |
| Methionine ox | ∆M_ox_ 147.0404 | ∆M_ox_ 73.520 | ∆M_ox_ 49.0135 |
| Phenylalanine | ∆F 147.0685 | ∆F 73.534 | ∆F 49.0228 |
| Arginine | ∆R 156.1011 | ∆R 78.050 | ∆R 52.0337 |
| Cysteine-cam | ∆C_cam_ 160.0092 | ∆C_cam_ 80.004 | ∆C_cam_ 53.3364 |
| Tyrosine | ∆Y 163.0633 | ∆Y 81.531 | ∆Y 54.3544 |
| tryptophan | ∆W 186.0793 | ∆W 93.039 | ∆W 62.0264 |

Cysteine-cam is carbamidomethylated cysteine prepared by treating reduced cysteine with iodoacetamide.
