# Supplementary material for: Evaluation of mass spectrometry MS/MS spectra for the presence of isopeptide crosslinked peptides
Source: PLoS One. 2021 Jul 9;16(7):e0254450. doi: 10.1371/journal.pone.0254450 (PMC8270460; doi:10.1371/journal.pone.0254450)
Supplement: S1 Fig — (DOCX) [file pone.0254450.s003.docx]

Evaluation of mass spectrometry MS/MS spectra for the presence of isopeptide crosslinked peptides

Lawrence M. Schopfer, Seda Onder, Oksana Lockridge

Eppley Institute, University of Nebraska Medical Center, Omaha, NE 68198 USA

Department of Biochemistry, School of Pharmacy, Hacettepe University, Ankara 06100, Turkey


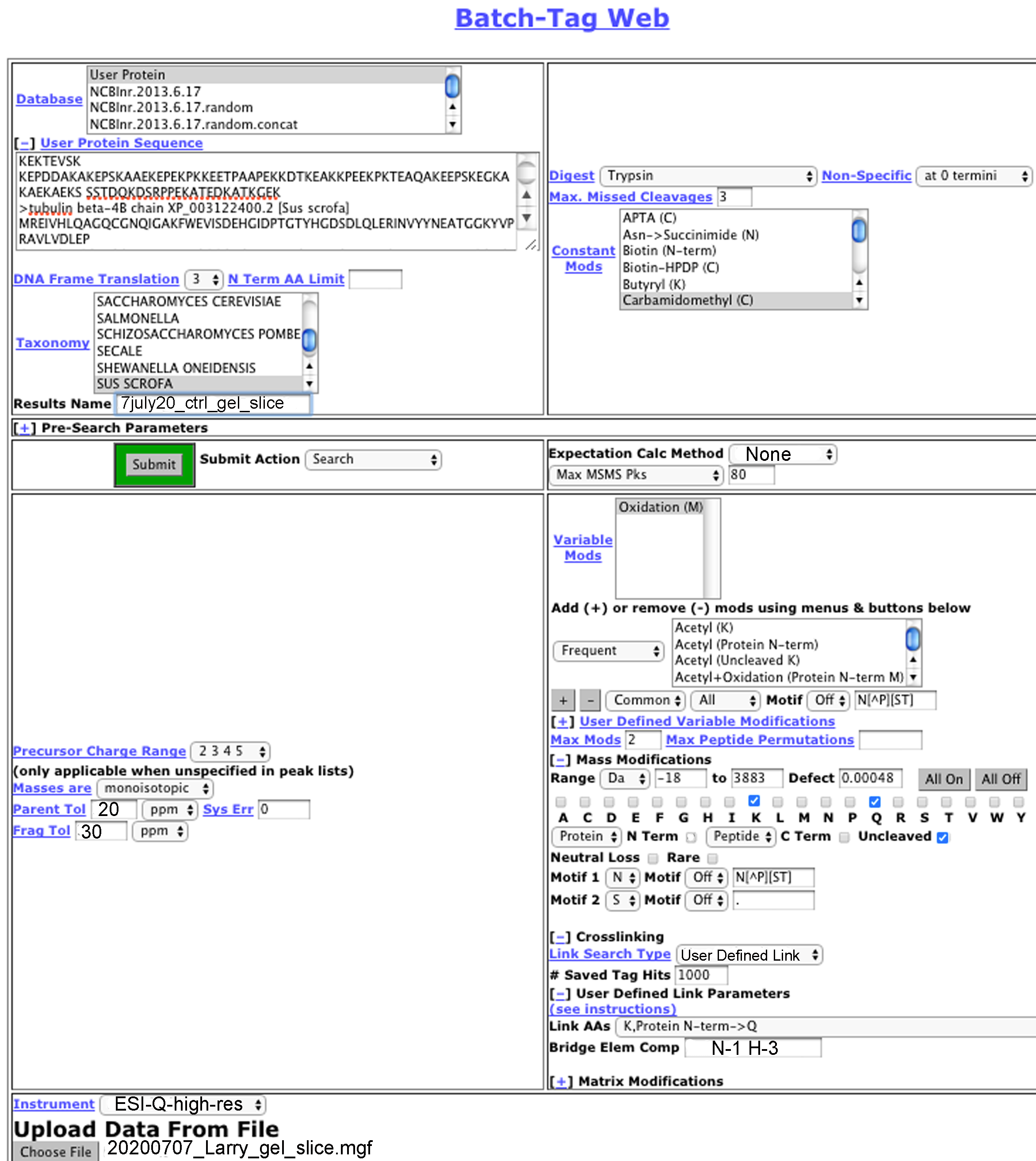


S1 Figure. Screen shot of Batch-Tag Web page in Protein Prospector. Crosslinks between lysine (K) and glutamine (Q) were identified in *.mgf files using the parameters shown on this page. The accession numbers and amino acid sequences of 6 proteins were pasted into the User Protein Sequence box.
