# Supplementary material for: Evaluation of mass spectrometry MS/MS spectra for the presence of isopeptide crosslinked peptides
Source: PLoS One. 2021 Jul 9;16(7):e0254450. doi: 10.1371/journal.pone.0254450 (PMC8270460; doi:10.1371/journal.pone.0254450)
Supplement: S2 Fig — (DOCX) [file pone.0254450.s004.docx]

Evaluation of mass spectrometry MS/MS spectra for the presence of isopeptide crosslinked peptides

Lawrence M. Schopfer, Seda Onder, Oksana Lockridge

Eppley Institute, University of Nebraska Medical Center, Omaha, NE 68198 USA

Department of Biochemistry, School of Pharmacy, Hacettepe University, Ankara 06100, Turkey

| 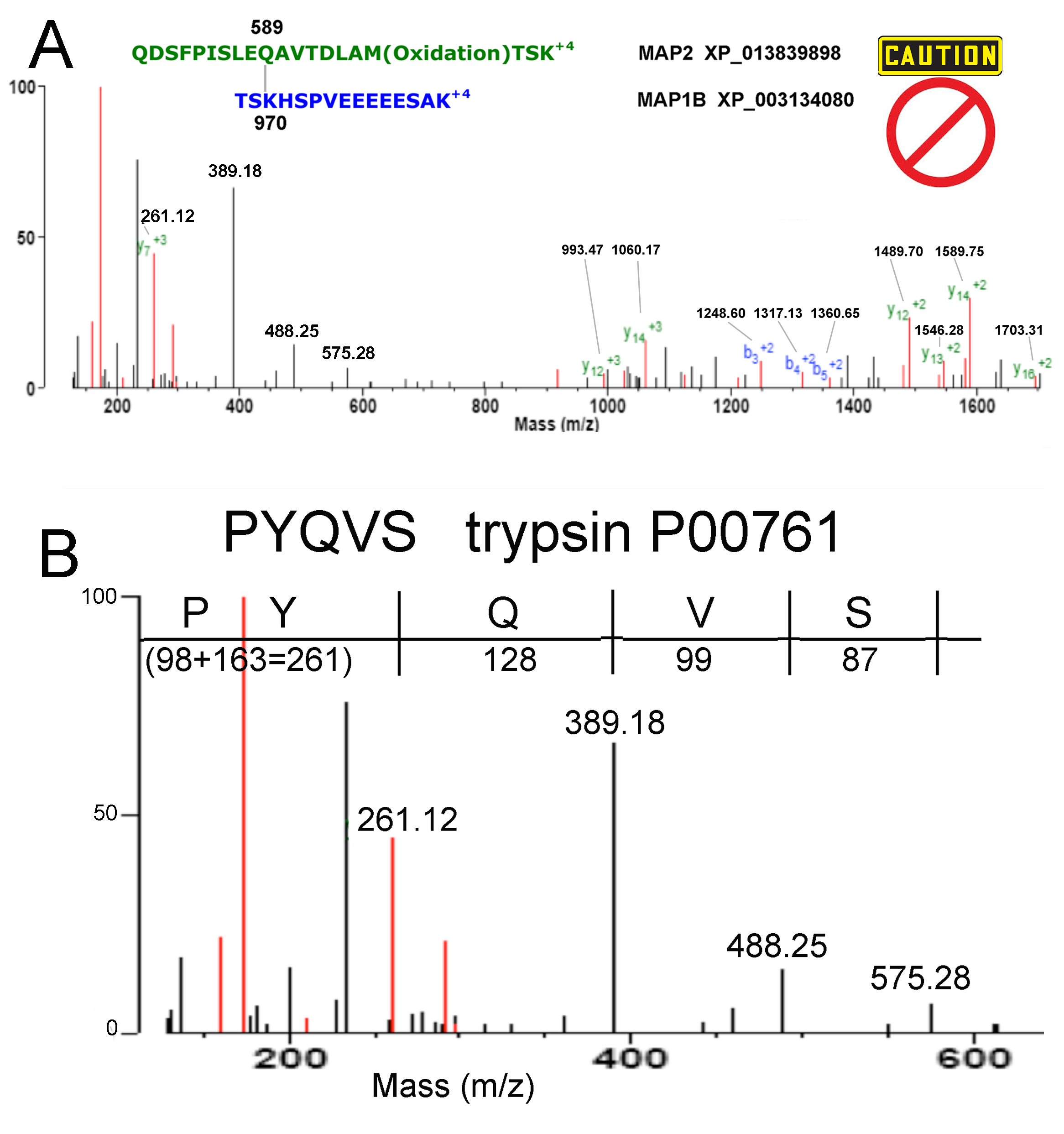 |
| --- |

S2 Figure. False positive crosslink. Panel A) MS/MS spectrum of a candidate crosslinked peptide pair identified by Protein Prospector. This apparent crosslink is actually a linear peptide of trypsin as demonstrated by manual evaluation. Panel B) Manual evaluation identified peaks in the 0 to 600 m/z range as charge +1 ions of trypsin. The list of mass differences in S2 Table was used to manually evaluate the MS/MS spectrum.
