# Supplementary material for: Evaluation of mass spectrometry MS/MS spectra for the presence of isopeptide crosslinked peptides
Source: PLoS One. 2021 Jul 9;16(7):e0254450. doi: 10.1371/journal.pone.0254450 (PMC8270460; doi:10.1371/journal.pone.0254450)
Supplement: S3 Fig — (DOCX) [file pone.0254450.s005.docx]

Evaluation of mass spectrometry MS/MS spectra for the presence of isopeptide crosslinked peptides

Lawrence M. Schopfer, Seda Onder, Oksana Lockridge

Eppley Institute, University of Nebraska Medical Center, Omaha, NE 68198 USA

Department of Biochemistry, School of Pharmacy, Hacettepe University, Ankara 06100, Turkey

| 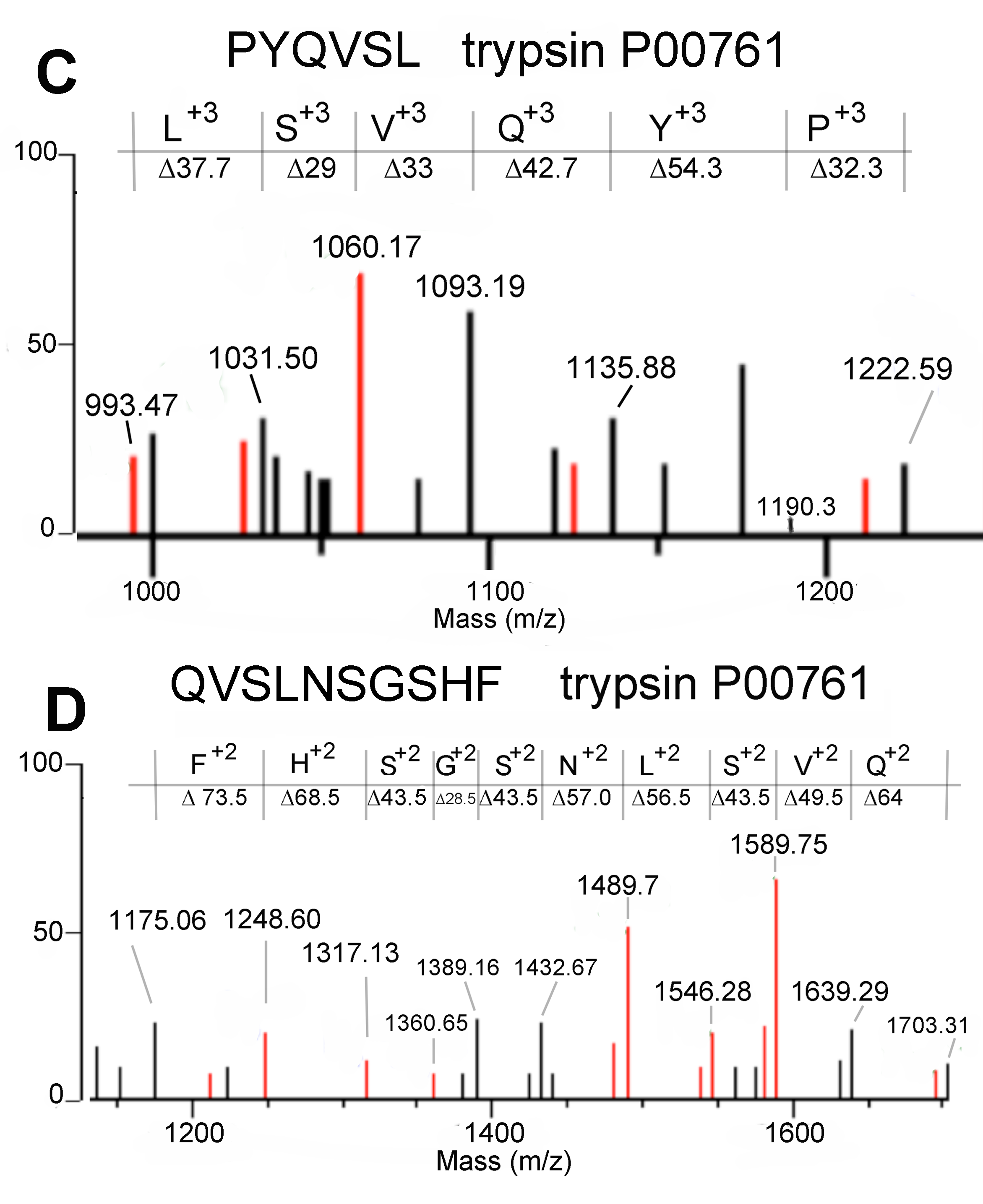 |
| --- |

S3 Figure. Panel C) Manual evaluation identified the charge state +3 ions in panel C as trypsin. Panel D) Manual evaluation identified the charge state +2 ions in panel D as trypsin.

The same manual evaluation strategy identified a series of charge state +3 ions belonging to trypsin in S3 Figure panel C, and a series of charge state +2 ions belonging to trypsin in S3 Figure panel D. We ruled out the possibility that the MS/MS spectrum in S2 Figure might represent both the MAP2 and MAP1B crosslink as well as trypsin, by finding that every y-ion and b-ion in S2 Figure panel A was assigned to trypsin in panels B, C and D. No y- or b- ions remained that exclusively fit the putative crosslinked peptide pair. We added a caution sign to S2 Figure panel A to make clear that this strongly supported example of an isopeptide crosslinked peptide is a false positive. The caution sign in S2 Figure panel A was adapted from a graphic by Iacobucci and Sinz (2017).[^1^](#_ENREF_1)

(1) Iacobucci, C., and Sinz, A. (2017) To Be or Not to Be? Five Guidelines to Avoid Misassignments in Cross-Linking/Mass Spectrometry. *Anal Chem* *89*, 7832-7835.
