# Supplementary material for: Evaluation of mass spectrometry MS/MS spectra for the presence of isopeptide crosslinked peptides
Source: PLoS One. 2021 Jul 9;16(7):e0254450. doi: 10.1371/journal.pone.0254450 (PMC8270460; doi:10.1371/journal.pone.0254450)
Supplement: S4 Fig — (DOCX) [file pone.0254450.s006.docx]

Evaluation of mass spectrometry MS/MS spectra for the presence of isopeptide crosslinked peptides

Lawrence M. Schopfer, Seda Onder, Oksana Lockridge

Eppley Institute, University of Nebraska Medical Center, Omaha, NE 68198 USA

Department of Biochemistry, School of Pharmacy, Hacettepe University, Ankara 06100, Turkey


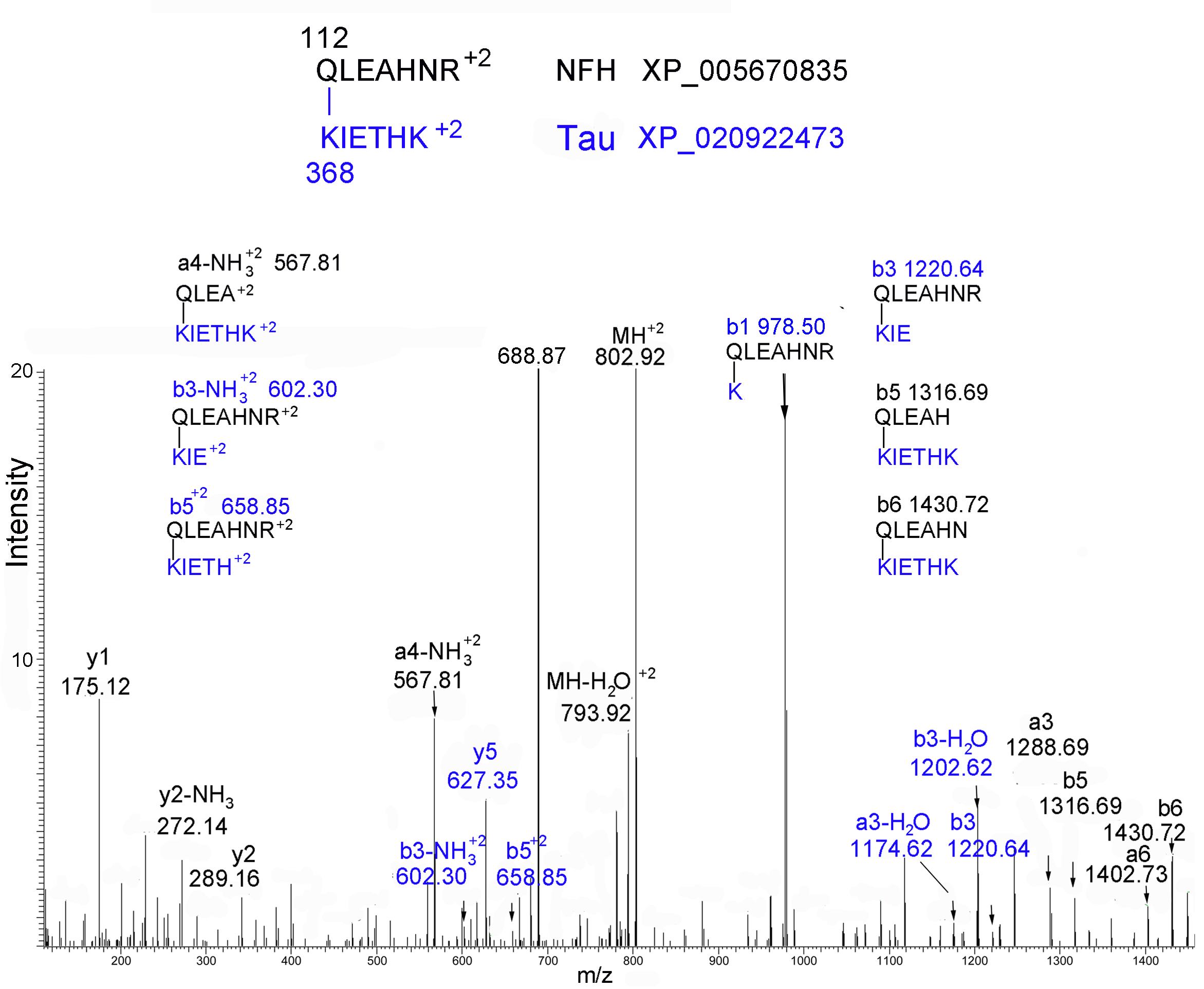


S4 Figure. MS/MS of the naturally-occurring isopeptide in MAP-rich tubulin. The crosslink between K368 of Tau and Q112 of NFH was identified in a tryptic digest of a MAP-rich tubulin gel slice. Arrows point to ten crosslink-specific ions that include ions from both peptides. Structures are shown for 7 crosslink-specific ions. The doubly-charged parent ion has a mass of 802.92 m/z.
