# Supplementary material for: Evaluation of mass spectrometry MS/MS spectra for the presence of isopeptide crosslinked peptides
Source: PLoS One. 2021 Jul 9;16(7):e0254450. doi: 10.1371/journal.pone.0254450 (PMC8270460; doi:10.1371/journal.pone.0254450)
Supplement: S1 Text — (DOCX) [file pone.0254450.s007.docx]

Evaluation of mass spectrometry MS/MS spectra for the presence of isopeptide crosslinked peptides

Lawrence M. Schopfer, Seda Onder, Oksana Lockridge

Eppley Institute, University of Nebraska Medical Center, Omaha, NE 68198 USA

Department of Biochemistry, School of Pharmacy, Hacettepe University, Ankara 06100, Turkey

**S1 Text. Manual evaluation protocol**

Manual evaluation is a critical step in the process of identifying crosslinked peptides. Manual evaluation starts with assigning a charge state to each peak in the MS/MS spectrum. This is accomplished by checking the isotopic splittings for each peak. A one mass unit interval indicates a singly-charged mass, a 0.5 mass unit interval indicates a doubly-charged mass, a 0.33 mass unit interval indicates a triply charged mass. The next step is to determine the difference between masses of identical charge state. An amino acid sequence is developed by matching the dehydro-masses for the various amino acids to the observed mass differences. For singly-charged masses the mass difference is simply the dehydro-mass. For doubly-charged masses the mass difference is one-half of the dehydro-mass, etc. Table S2 lists the mass differences for dehydro-amino acid residues in charge states +1, +2, and +3. Identification of a sequence is facilitated by foreknowledge of the crosslinked sequence provided by Protein Prospector.

A complication develops when considering the masses of the terminal amino acids. The mass for a C-terminal amino acid from a y-series is 19 mass units larger than the dehydro-mass, e.g. the dehydro-mass of lysine is 128 Da while the mass for a C-terminal lysine from a y-series is 147 Da. The C-terminal of a y-series contains only one amino acid and is therefore the smallest mass. The mass for an N-terminal amino acid from a y series is equal to the dehydro-mass. The mass for a C-terminal amino acid from a b-series is 18 mass units larger than the dehydro-mass, e.g. the dehydro-mass of tyrosine is 163 Da while the mass for a C-terminal tyrosine from a b-series is 181 Da. The mass for an N-terminal amino acid from a b-series is 1 mass unit larger than the dehydro-mass, e.g. the dehydro-mass for glycine is 57 Da while the mass of the N-terminal glycine is 58 Da. The N-terminal of a b-series is the first amino acid in the sequence and therefore the smallest mass. A list of dehydro-masses can be found in various publications [^1-3^](#_ENREF_1). We find that manual evaluation of the MS/MS spectrum for a crosslinked peptide commonly reveals peaks that are not identified by Protein Prospector. In some cases, these masses are fragments of the crosslinked peptide that Protein Prospector did not identify. In other cases, the extra peaks can be assigned to a contaminating peptide coming from another protein, or the extra peaks fit a sequence that does not match any protein in the NCBI or UniProt databases. Such unassigned peaks can be present even when the analyte appears to be a single pure protein. In extreme cases, a well-supported crosslinked spectrum is best fit to a non‑crosslinked peptide, making it a false positive.

A striking example of a false positive MS/MS spectrum is shown in S2 Figure panel A. This shows an MS/MS spectrum that has strong support for a crosslink between MAP2 and MAP1B. Fragment ions from both peptides are present. There are 16 crosslink specific ions. Crosslink specific ions are present as a series of related masses b3^+2^, b4^+2^, b5^+2^ and y12^+2^, y13^+2^, y14^+2^, y16^+2^. The matched intensity is 44.4% with score 26.1 and score difference 0.8.

However, when we manually evaluated the spectrum in panel A, we came to the conclusion that it represents a peptide from trypsin. S2 Figure panel B identifies the +1 charge state ions from trypsin as peaks in S2 Figure panel A. S3 Figure panels C and D identify the +3 charge state and +2 charge state ions of trypsin as peaks in S2 Figure panel A. Every y-ion and b-ion in S2 Figure panel A was assigned to trypsin in panels B, C and D. No y- or b- ions remained that exclusively fit the putative crosslinked peptide pair. We added a caution sign to S2 Figure panel A to make clear that this example of an apparent isopeptide crosslinked peptide is a false positive.
