# Supplementary material for: Evaluation of mass spectrometry MS/MS spectra for the presence of isopeptide crosslinked peptides
Source: PLoS One. 2021 Jul 9;16(7):e0254450. doi: 10.1371/journal.pone.0254450 (PMC8270460; doi:10.1371/journal.pone.0254450)
Supplement: S1 Raw image — (DOCX) [file pone.0254450.s008.docx]

S1-raw_image for

PONE-D-21-10112R1
Evaluation of mass spectrometry MS/MS spectra for the presence of isopeptide crosslinked peptides

Corresponding author Oksana Lockridge [olockrid@unmc.edu](mailto:olockrid@unmc.edu)


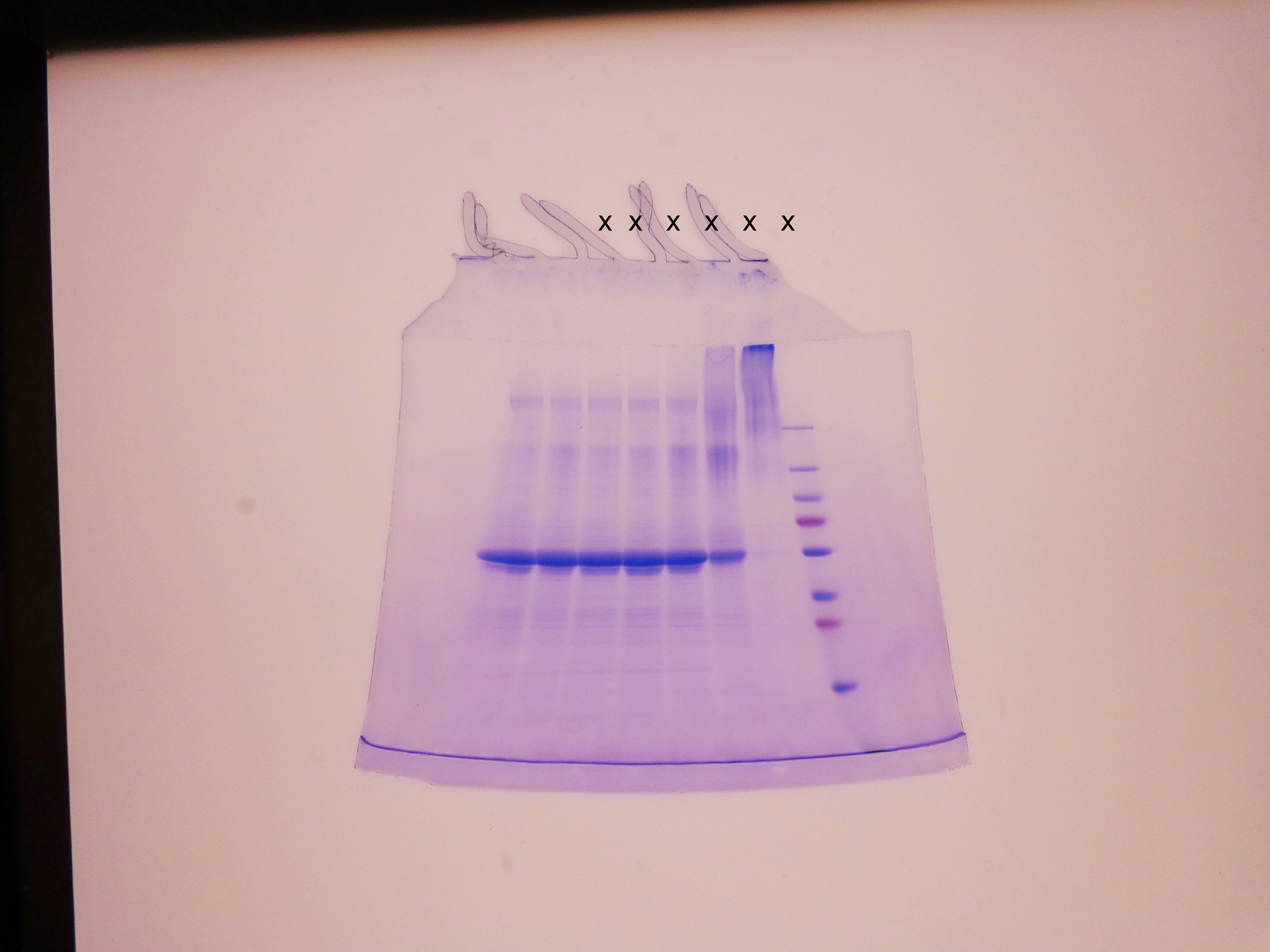


SDS gel stained with Coomassie blue. Image taken with a Sony camera. Lanes marked with an X were deleted from Figure 2 in our manuscript.


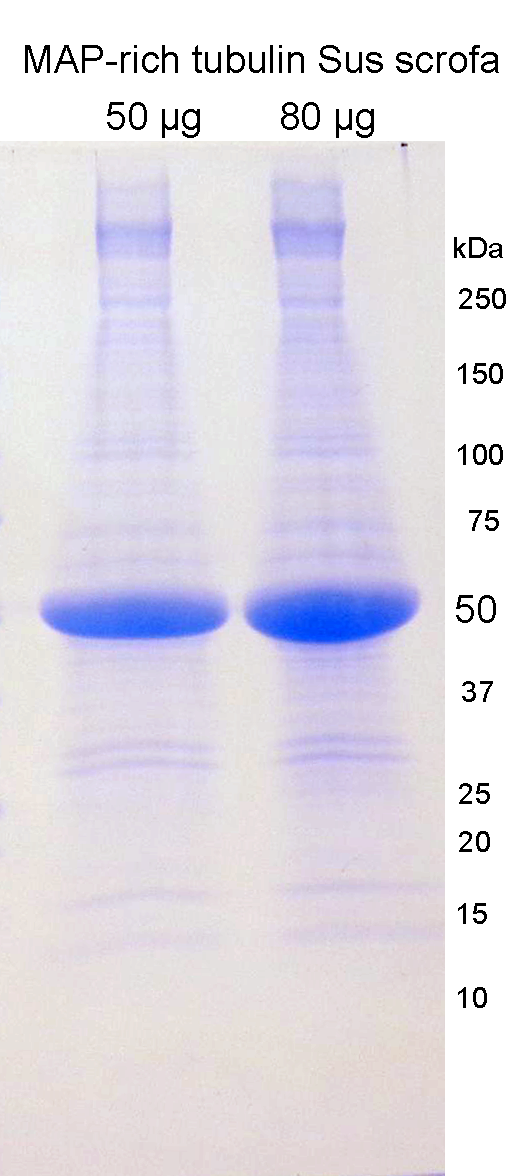


Figure 2 in PONE-D-21-10112R12
